# Integrating in vitro data and physiologically based kinetic modeling-facilitated reverse dosimetry to predict human cardiotoxicity of methadone

**DOI:** 10.1007/s00204-020-02766-7

**Published:** 2020-05-04

**Authors:** Miaoying Shi, Hans Bouwmeester, Ivonne M. C. M. Rietjens, Marije Strikwold

**Affiliations:** 1 Division of Toxicology, Wageningen University, Stippeneng 4, 6708 WE Wageningen, The Netherlands; 2 Van Hall Larenstein University of Applied Sciences, 8901 BV Leeuwarden, The Netherlands

**Keywords:** Cardiac electrophysiology, Methadone, Human induced pluripotent stem cell-derived cardiomyocytes (hiPSC-CM), Quantitative in vitro to in vivo extrapolation (QIVIVE), Physiologically based kinetic (PBK) modeling, Reverse dosimetry

## Abstract

Development of novel testing strategies to detect adverse human health effects is of interest to replace in vivo-based drug and chemical safety testing. The aim of the present study was to investigate whether physiologically based kinetic (PBK) modeling-facilitated conversion of in vitro toxicity data is an adequate approach to predict in vivo cardiotoxicity in humans. To enable evaluation of predictions made, methadone was selected as the model compound, being a compound for which data on both kinetics and cardiotoxicity in humans are available. A PBK model for methadone in humans was developed and evaluated against available kinetic data presenting an adequate match. Use of the developed PBK model to convert concentration–response curves for the effect of methadone on human-induced pluripotent stem cell-derived cardiomyocytes (hiPSC-CM) in the so-called multi electrode array (MEA) assay resulted in predictions for in vivo dose–response curves for methadone-induced cardiotoxicity that matched the available in vivo data. The results also revealed differences in protein plasma binding of methadone to be a potential factor underlying variation between individuals with respect to sensitivity towards the cardiotoxic effects of methadone. The present study provides a proof-of-principle of using PBK modeling-based reverse dosimetry of in vitro data for the prediction of cardiotoxicity in humans, providing a novel testing strategy in cardiac safety studies.

**Electronic supplementary material:**

The online version of this article (10.1007/s00204-020-02766-7) contains supplementary material, which is available to authorized users.

## Introduction

Traditional approaches for the risk and safety assessment of compounds rely heavily on toxicity data derived from laboratory animals, which are gradually being recognized as inappropriate models for the prediction of human health effects due to toxicodynamic and toxicokinetic differences between animals and human (Ewart et al. 2014; Pang et al. 2019). This consideration as well as the fact that animal-based testing strategies are cost and labor intensive, while also increasingly considered unethical, has stimulated the development of novel testing strategies, leading to a paradigm shift in toxicity testing (Judson et al. 2014). Novel testing strategies generally apply in vitro assays and take into account insight in the modes of action underlying the toxicity (Bernauer et al. 2005). However, in vitro assays provide hazard information and concentration–response curves that require translation to corresponding human dose–response curves, taking into account human toxicokinetics, to enable their use in human risk and safety assessment of compounds (Bell et al. 2018; Blaauboer 2010).

Over the last decade, several proof-of-principle studies indicated that combining in vitro toxicity assays with physiologically based kinetic (PBK) modeling, which describes the absorption, distribution, metabolism and excretion (ADME) of a compound in a defined species, can adequately predict in vivo dose–response curves (Louisse et al. 2017; Rietjens et al. 2011). For example, quantitative in vitro to in vivo extrapolation (QIVIVE) using PBK modeling-based reverse dosimetry was shown to adequately predict the in vivo toxicity for different endpoints, including developmental toxicity (Li et al. 2017; Louisse et al. 2010; Strikwold et al. 2013, 2017), liver toxicity (Ning et al. 2017), nephrotoxicity (Abdullah et al. 2016) and neurotoxicity (Zhao et al. 2019). To further explore the potential applicability of this in vitro–in silico approach, the aim of the present study was to investigate whether the PBK modeling-based reverse dosimetry can be extended to predict in vivo cardiotoxicity in human, thereby providing a novel testing strategy for cardiac safety testing.

Cardiotoxicity is an important endpoint in pharmaceutical safety testing and has been a leading cause of drug attrition in preclinical drug development (Pang et al. 2019; Stevens and Baker 2009). In addition, cardiotoxicity is also a relevant endpoint in food safety, given that many food-borne alkaloids from botanicals and botanical preparations, including, for example, synephrine from bitter orange (*Citrus aurantium*) and nuciferine from lotus (*Nelumbo nucifera*), raise a concern with respect to potential cardiotoxicity (Kratz et al. 2017). Potential cardiotoxicity includes functional and structural disruption of the cardiovascular system by interfering with ion channels, intracellular organelles and cellular signaling pathways (Clements et al. 2015; Pang et al. 2019). Particularly, cardiac electrophysiological alterations such as delayed ventricular repolarization are endpoints of interest for cardiac safety assessment. Delayed ventricular repolarization can result in a prolonged QTc interval (time from ventricular depolarization and repolarization corrected for heart rate) in the electrocardiogram (ECG) which is associated with increased risk of arrhythmia including polymorphic ventricular tachyarrhythmia (torsade de pointes, Tdp) (Ewart et al. 2012; Harris et al. 2013; Kannankeril et al. 2010; Redfern et al. 2003; Wakefield et al. 2002). Current regulatory guidelines to evaluate in vitro electrophysiological cardiotoxicity are based on ion channel inhibition assays using cell lines transfected with specific ion channels, including especially human ether-à-go-go-related gene (hERG) channels which play a critical role in cardiac repolarization (ICH 2005a; Martin et al. 2004; Zwartsen et al. 2019). However, such an approach focussing on a single type of ion channel fails to address effects induced on other channels (Mirams et al. 2011; Rehnelt et al. 2017). Recently, human-induced pluripotent stem cell-derived cardiomyocytes (hiPSC-CM) have been reported to provide a physiological relevant in vitro model for human cardiotoxicity testing. These hiPSC-CM express major cardiac ion channels and show typical electrophysiological responses upon the exposure to compounds (Garg et al. 2018; Ma et al. 2011). In the present study, hiPSC-CM were applied in combination with the multi electrode array (MEA) technique measuring the extracellular field potential of electrically active cardiomyocytes, which is considered a promising tool to assess electrophysiological alteration and arrhythmias (Ando et al. 2017; Harris et al. 2013; Kitaguchi et al. 2017; Li et al. 2016). The parameters obtained from extracellular field potential waveforms are considered to resemble the parameters observed in the human ECG (Zwartsen et al. 2019), which allows use of the hiPSC-CM MEA assay as an adequate in vitro model for QIVIVE.

**Fig. 1:**
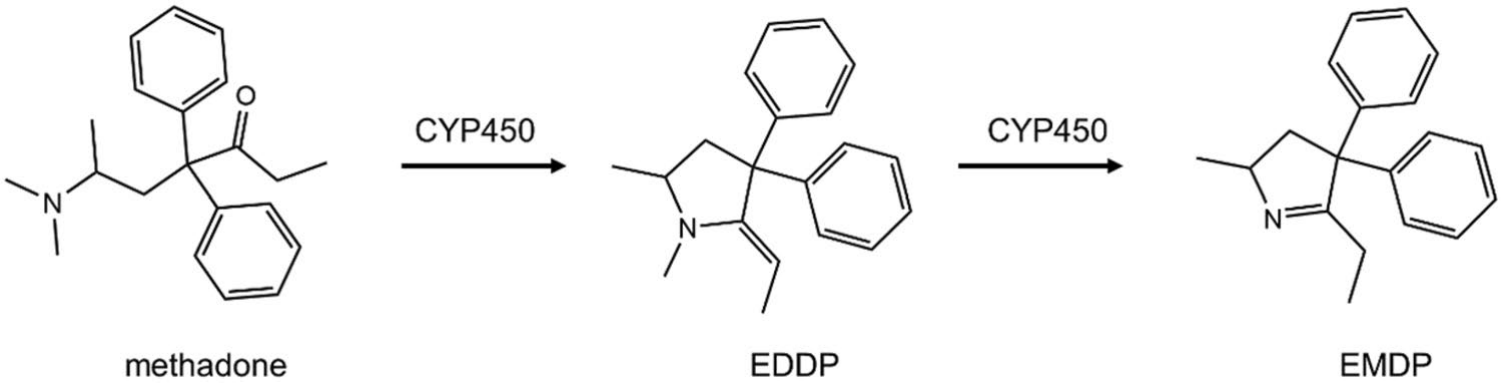
Metabolic conversion of methadone to 2-ethylidene-1,5-dimethyl-3,3-diphenylpyrrolidine (EDDP), and 2-ethyl-5-methyl-3,3-diphenylpyrroline (EMDP) by cytochrome P450 (CYP450)

The model compound selected for the present study was methadone (Fig. 1). Methadone is a synthetic drug for the treatment of opioid dependence and chronic pain. Methadone is metabolized by cytochromes P450 (CYP) mainly in the liver (Eap et al. 2002; Nilsson et al. 1982). The primary metabolite, 2-ethylidene-1,5-dimethyl-3,3-diphenylpyrrolidine (EDDP), is formed via *N*-demethylation and cyclisation, and a subsequent *N*-demethylation leads to the secondary metabolite, 2-ethyl-5-methyl-3,3-diphenylpyrroline (EMDP) (Fig. 1). Methadone has been reported to cause cardiotoxic side effects in human clinical studies in which prolonged QTc interval and TdP have been observed in subjects receiving methadone maintenance treatment (Alinejad et al. 2015; Eap et al. 2002; Justo et al. 2006). Several in vitro studies using electrophysiological-based patch clamp demonstrated an association between the cardiotoxicity of methadone and the inhibition of hERG channels (Eap et al. 2007; Kuryshev et al. 2010).

In the present study, the in vitro concentration-dependent cardiotoxicity of methadone and its metabolites EDDP and EMDP was quantified in hiPSC-CM using the MEA technique. Additionally, a PBK model for methadone kinetics in human was developed by integrating data from literature as well as experimentally obtained metabolic parameters. This PBK model was subsequently used to translate the in vitro toxicity data to predict in vivo cardiotoxicity in human. The data thus obtained were compared to available data on the effect of methadone on cardiac parameters in subjects that received methadone maintenance treatment.

## Materials and methods

### Chemical and biological materials

Methadone hydrochloride (≥ 98%), EDDP perchlorate (≥ 98%), EMDP hydrochloride solution (1.0 mg/ml), Tris (hydroxymethyl) aminomethane (Trizma^®^ base), ammonium formate and fibronectin were purchased from Sigma-Aldrich (Zwijndrecht, The Netherlands). Methadone and metabolites were ordered under the opium exemption license number 104783 03 WCO, which is registered at Farmatec (executive organization of the Ministry of Health, Welfare and Sport, The Hague, The Netherlands). Dimethyl sulfoxide (DMSO, 99.7%) was obtained from Merck (Schiphol-Rijk, The Netherlands). Phosphate-buffered saline (PBS) was purchased from Gibco (Paisley, Scotland, UK). Acetonitrile (UPLC/MS grade) was obtained from Biosolve BV (Valkenswaard, The Netherlands). hiPSC-CM (Pluricyte^®^ Cardiomyocytes, cat# PCMI-1031-1, lot# 60151) and Pluricyte^®^ Cardiomyocyte medium were obtained from Ncardia (Leiden, The Netherlands). Pooled human liver microsomes (from 150 donors), pooled human intestinal microsomes (from 7 donors) and reduced nicotinamide adenine dinucleotide phosphate (NADPH) regenerating system solution A and solution B were purchased from Corning (Woburn, MA, USA). Pooled human plasma and rapid equilibrium dialysis (RED) materials, including RED inserts, RED base plates and sealing tape were obtained from Thermo Fisher Scientific (Bleiswijk, The Netherlands).

### General outline of the PBK modeling‑based reverse dosimetry approach

The PBK modeling-based reverse dosimetry approach to predict the in vivo dose–response curves from in vitro cardiotoxicity concentration–response data included the following steps: (1) establishment of the in vitro concentration–response curves for methadone and its metabolites EDDP and EMDP in hiPSC-CM using the MEA, (2) development of a PBK model for methadone and its metabolites in human using metabolic parameters obtained from in vitro incubations with pooled human liver microsomes, and parameters derived from in silico simulations and the literature, (3) evaluation of the PBK model, (4) translation of in vitro concentration–response curves to in vivo dose–response curves using the PBK model, and (5) evaluation of the PBK modeling-based reverse dosimetry approach by comparing predicted dose–response data to data obtained from literature on the effect of methadone on cardiac parameters in subjects receiving methadone maintenance treatment.

### In vitro cardiotoxicity of methadone and metabolites in hiPSC-CM using the MEA

The MEA system of Multi Channel System (MCS GmbH, Ruetlingen, Germany) combined with Pluricyte^®^ Cardiomyocytes was used to detect the cardiotoxicity of methadone, and the metabolites EDDP and EMDP. The Pluricyte^®^ Cardiomyocytes were thawed and seeded on the six-well MEA chips (60-6well MEA200/30iR-Ti-tcr, MCS GmbH) according to the manufacturer’s protocol. Briefly, each well of the MEA chips was precoated with 50 μg/ml fibronectin for 3 h in the incubator at 37 °C with 5% CO_2_. The fibronectin coating solution was aspirated before seeding. Cells were thawed in the incubator at 37 °C for exactly 4 min and carefully transferred to a 50-ml tube. The original vial was rinsed with serum free Pluricyte^®^ Cardiomyocyte Medium and added drop-wise to the tube containing the cardiomyocytes. Subsequently, cell counting was manually performed, using 20 μl of obtained homogenous cell suspension in a Buerker-Tuerk Counting Chamber (Marienfeld Superior GmbH & Co. KG, Lauda-Königshofen, Germany) and at the same time the remaining cells were centrifuged at 300*g* for 3 min. Then, the supernatant was removed and medium was drop-wisely added to reach the aimed concentration of cells in the suspension (10^4^ cells/μl). 2-μl cell suspension per well was placed on the six-well MEA chips in a density of 10^4^ cells/μl. After 3-h incubation (37 °C, 5% CO_2_), 200 μl of medium was filled into each well of the MEA chips which were subsequently incubated at 37 °C with 5% CO_2_ and refreshed with medium every 2 days.

**Fig. 2:**
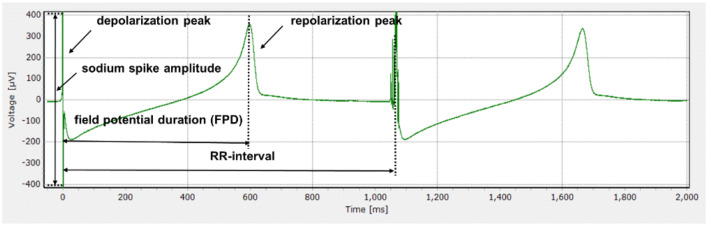
Typical extracellular field potential waveform consisting of a rapid upstroke corresponding to depolarization, a slow wave/plateau and a repolarization peak. Signals were generated under the baseline condition (0.1% (v/v) DMSO) in Pluricyte cardiomyocytes cultured in six-well MEA-chips measured by the MEA2100-System platform of MCS

At 7–8 days after seeding, MEA chips were placed on the headstage of a MEA2100-system (MCS GmbH) integrated with the chamber providing a stable atmosphere (37 °C, 5% CO_2_) to record the extracellular field potential (Fig. 2) of spontaneous beating hiPSC-CM. After an equilibration time of 20 min, half of the medium (100 μl) in each well was replaced by culture medium containing 0.2% (v/v) DMSO to reach a final concentration of 0.1% (v/v) DMSO, which was used as baseline condition. Subsequently, the model compounds were tested in separate wells, and each test compound was cumulatively added to the well with increasing concentrations in the same way (Harris et al. 2013; Nozaki et al. 2017; Ando et al. 2017). At each concentration, the extracellular field potential was recorded for 1 min after 10-min exposure. Stock solutions of model compounds were prepared in DMSO and further diluted in Pluricyte^®^ Cardiomyocyte medium to make exposure medium with the final concentration of 0.1% (v/v) DMSO. The following concentrations were tested, 0.01, 0.03, 0.1, 0.2, 0.3, 0.4, 1, 3, 10, 30 µM (methadone), 0.01, 0.03, 0.1, 0.3, 1, 3, 10, 30 µM (EDDP) and 0.1, 0.3, 1, 3, 10, 30 µM (EMDP), at which no cytotoxicity was observed (data not shown). The test concentrations of methadone were based on reported human methadone plasma concentrations that were observed after oral methadone treatment. Same test concentrations were chosen for EDDP and EMDP, which enables definition of concentration-dependent curves for EDDP and EMDP that allow potency comparison.

One well of 0.1% (v/v) DMSO on each MEA chip was used as the vehicle control well and run at the same time as the compound exposure wells to correct for time- and DMSO-dependent effects on the field potential. A detailed exposure scheme can be found in Fig. S1. Data were collected using Cardio 2D software (MCS GmbH) with a sample frequency of 10 kHz and a 0.1–3.5-kHz band-pass filter.

After exposure, MEA data generated from the electrodes showing stable baseline field potential with clearly visible depolarization (peak amplitude ≥ 200 μV) and repolarization peaks (peak amplitude ≥ 20 μV) (Ando et al. 2017; Sala et al. 2017) were selected for further analysis using Multiwell-Analyzer software Version 1.5.1.0 (MCS GmbH). Field potential duration (FPD) was defined as duration between the beginning of the sodium spike and the repolarizing peak (Fig. 2). RR-intervals were defined as the duration between two depolarization peaks (Fig. 2). The FPD and RR-interval were measured as the average of at least 30 beats from 1-min recording at each concentration of the test compound. In addition, the Fridericia formula (Eq. 1) was applied to correct for the effect of beat rate on FPD (Vandenberk et al. 2016) as widely used in other MEA studies (Ando et al. 2017; Kitaguchi et al. 2017):

1 $${\text{FPDc}} = \frac{{{\text{FPD}}}}{{\sqrt[3]{{{\text{RR}}\;{\text{interval}}}}}}.$$

In this formula, the FPD and RR-interval are expressed in seconds. Data were collected from at least three independent experiments (4–8 wells, 26–38 electrodes), using a new vial of cells (all from the same batch) at each independent experiment. In vitro cardiotoxic effects are expressed as relative percentage of FPDc compared to the FPDc results obtained for the baseline condition [0.1% (v/v) DMSO] and further corrected for the time- and DMSO-dependent effects by subtracting the response of 0.1% (v/v) DMSO obtained from the corresponding time-matched vehicle control well. The concentrations inducing irregularities in the field potential trace (Fig. S2) were also noted. Such irregularities included arrhythmia-type changes in the waveform, a flattened unclear second peak and/or beating arrest (Asakura et al. 2015; Kitaguchi et al. 2017; Nakamura et al. 2014; Zwartsen et al. 2019). Concentrations inducing these irregularities were excluded from the FPD analysis since the FPD and RR-interval could not be determined.

Effective concentrations expressed as the FPDc (% to the baseline control) were determined using the benchmark dose (BMD) approach which was performed as described in the “Evaluation of the PBK modeling-based reverse dosimetry approach”. The concentration–response curves were plotted with GraphPad Prism 5.0 using the four-parameters logistic fit (GraphPad Software Inc., San Diego, USA). Each data point is presented as the mean value of at least three independent experiments ± standard deviation (SD). Statistical significance of the changes in response of cells exposed to the compound compared to the solvent control was analyzed by one-way ANOVA followed by post Dunnett test. Values of *p* < 0.05 were regarded as statistically significant (*p* < 0.05: *, *p* < 0.01: ** and *p* < 0.001: ***). Statistical analysis was performed by GraphPad Prism 5.0 (GraphPad Software Inc.).

### In vitro microsomal incubations

In vitro incubations were performed to obtain the kinetic parameters for the conversion of methadone by human liver microsomes. To this end, incubation conditions were optimized to obtain linear reaction rates with respect to microsomal protein levels (0.1–2 mg/ml protein) and incubation time (1–120 min) at 50-µM methadone. The final incubation mixtures (final volume of 160 µl) consisted of 0.1-M Tris–HCl (pH 7.4–7.5), NADPH regeneration system (final concentrations 1.3-mM NADP^+^, 3.3-mM glucose-6-phosphate, 0.4-U/ml glucose-6-phosphate dehydrogenase and 3.3-mM magnesium chloride) and methadone at eight final concentrations ranging from 10 to 1500 µM diluted from a 100-mM stock solution in water. The test concentrations were chosen to enable adequate analysis of Michaelis–Menten kinetics. After one-min pre-incubation of this solution at 37 °C, the reactions were initiated by addition of human liver microsomes giving a final concentration of 0.5 mg/ml microsomal protein and incubations were performed in a shaking water bath at 37 °C for 40 min. Control incubations were performed in the absence of NADPH which was replaced with Tris–HCl. The reactions were terminated by addition of 40-µl ice-cold acetonitrile. Samples were kept on ice for at least 20 min and then centrifuged at 18,000*g* for 5 min at 4 °C to precipitate microsomal proteins. The supernatant was collected for the quantification of EDDP formation, which was analyzed by Ultra-Performance Liquid Chromatography PhotoDiode Array (UPLC-PDA, Waters) as described in the “Quantification of methadone and its metabolites by UPLC–PDA analysis” section.

The formation of the secondary metabolite EMDP from EDDP was investigated by incubating 1000-µM EDDP under the same conditions as described above for the microsomal methadone incubations. The kinetic parameters for the conversion of methadone by intestinal microsomes were determined under the same conditions as the incubations with liver microsomes after the incubation conditions were optimized with respect to microsomal protein levels (0.1–2 mg/ml protein) and incubation time (1–120 min) at 50-µM methadone.

The apparent maximum velocity (*V*_max_) and the apparent Michaelis–Menten constant (*K*_m_) describing the conversion of methadone to EDDP were determined using the Michaelis–Menten Eq. (2):

2 $$v = \frac{{V_{{\max}} \times \left[ S \right]}}{{K_{{\text{m}}} + \left[ S \right]}},$$

where [*S*] is the substrate concentration (µM) and *v* is the rate of EDDP formation (nmol/min/mg protein). *V*_max_ and *K*_m_ were obtained by fitting the data to Eq. (2) in GraphPad Prism 5.0 (GraphPad Software Inc.). Data were collected from three independent experiments and each data point is presented as the mean value ± SD.

### Determination of unbound fraction of methadone and EDDP in in vitro hiPSC-CM MEA assay medium and in human plasma

The rapid equilibrium dialysis (RED) assay was performed to determine the unbound fraction (*f*_u_) of methadone and EDDP in in vitro medium and in pooled human plasma using the protocol adapted from the manufacturer of the RED device (Thermo Fisher Scientific 2017). In short, methadone or EDDP was added to the in vitro medium or pooled human plasma to reach a concentration of 150 µM in test sample solution and PBS was used as buffer. 300-µl test sample solution and 500-µl PBS were, respectively, added to the sample chamber and the buffer chamber of the RED insert, which was subsequently incubated for 5 h at 37 °C at 250 rpm on an orbital shaker to reach equilibrium (van Liempd et al. 2011). Then, 25 µl of post-dialysis samples were collected from the sample chambers and transferred to test sample tubes followed by an addition of 25 µl PBS. Equal volumes of post-dialysis samples collected from the buffer chamber which were then mixed with 25 µl of in vitro medium or human plasma in the buffer sample tubes. Then, both samples were precipitated using 300-µl cold acetonitrile/water (90/10 v/v). The samples were put on ice for 30 min followed by centrifugation for 30 min at 15,000*g*. Then, supernatants were collected for UPLC–PDA analysis. The fraction unbound was calculated with Eq. (3) (van Liempd et al. 2011; Waters et al. 2008):

3 $$f_{{\text{u}}} = { }\frac{{\text{concentration in buffer chamer }}}{{\text{concentration in sample chamer}}}$$

The measurements were performed in triplicate in two independent experiments.

### Quantification of methadone and its metabolites by UPLC–PDA analysis

The quantification of methadone and its metabolites was performed by UPLC–PDA analysis using a Waters Acquity UPLC H_class system (Etten-Leur, The Netherlands) equipped with a Waters Acquity BEH C18 (1.7 µm, 2.1 × 50 mm) column. For optimal separation, a gradient of 20-mM ammonium formate (pH = 5.7) (solvent A) and acetonitrile (solvent B) with a flow rate of 0.3 ml/min was applied as follows: the initial condition was 90:10 (A:B); then the gradient was increased linear to 98% B over 8 min; then set to the initial conditions in 2 min and re-equilibrated for 5 min. Retention times of methadone, EDDP and EMDP were 4.7, 4.4 and 6.3 min, respectively. Identification of methadone and its metabolites was based on comparison of their retention time and UV spectrum to those of commercially available reference compounds. Quantification was based on comparison of the respective peak areas to the peak areas of corresponding calibration curves which were prepared using the reference compounds (*R*^2^ > 0.999).

### Establishment of the PBK model for methadone and EDDP

**Fig. 3:**
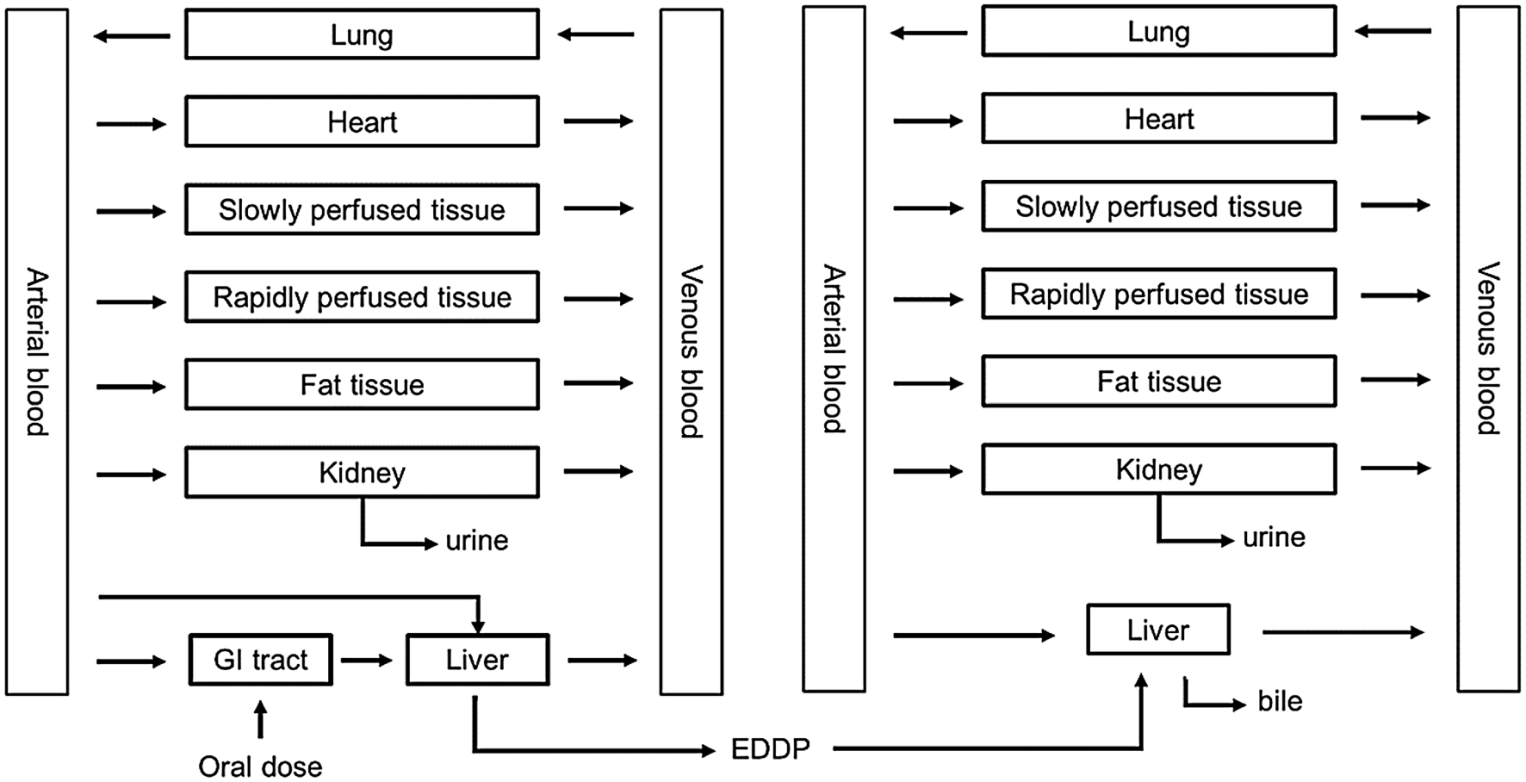
Schematic diagram of the PBK model of methadone including a submodel for EDDP

In the present paper, a PBK model describing the ADME of methadone and its major metabolites in human was developed. Figure 3 presents the schematic diagram of the PBK model including a submodel for the major metabolite EDDP and the compartments relevant for the ADME characteristics of methadone and EDDP. A submodel for EDDP was included to enable the prediction of internal concentrations of EDDP required to evaluate if EDDP will be formed in quantities that are relevant for cardiotoxicity. Considering that methadone is usually administered to the opioid dependent population or patients with chronic pain on a daily basis, a PBK model for repeated dosing of methadone was developed.

The absorption rate constant (ka) and fraction absorbed (Fa) are two key parameters describing the absorption of methadone. The uptake of methadone from the gastrointestinal (GI) tract was reported to follow a first-order process (Yang et al. 2006) with a mean ka value of 0.59/h obtained from several studies (Foster et al. 2000; Wolff et al. 2000). A mean Fa value of 0.88 was reported by Ke et al. (2014).

To describe the distribution, tissue: blood partition coefficients (*P*) of methadone and EDDP were obtained by dividing tissue: plasma partition coefficients by the corresponding blood/plasma ratio (BPr) obtained from subjects on methadone maintenance treatment (Hsu et al. 2013), to correct for the differences in the distribution of the compounds in blood and plasma. The tissue: plasma partition coefficients of methadone and EDDP were calculated using prediction method 1 which applies the algorithms of Berezhkovskiy (2004) in the Simcyp Simulator V18 Release 1 (Certara, Sheffield, UK) requiring information on the fraction unbound in plasma (*f*_u,p_), lipophilicity (logP) and acid–base properties (pKa). The logP and pKa values of methadone were obtained from literature (Gerber et al. 2001; Ke et al. 2014). The logP and pKa of EDDP were obtained from Marvinsketch (ChemAxon, Hungary). The *f*_u,p_ of methadone was obtained from the in silico Simcyp prediction tool (Certara). The *f*_u,p_ value of 0.3 for EDDP was obtained from the study of Moody et al. (2008). The *f*_u,p_ of methadone and EDDP were also measured using pooled human plasma in the current study (see “RED assay” section). Since the influence of blood: tissue partition coefficients derived based on different *f*_u,p_ values on the model output was negligible (data not shown), the blood: tissue partition coefficients calculated with the Simcyp-derived *f*_u,p_ were used.

Liver was identified as the metabolizing organ in the PBK model since conversion of methadone was reported to primarily occur in the liver (Foster et al. 2004; Totah et al. 2008). Although Oda and Kharasch (2001) observed conversion of methadone in in vitro human intestinal microsomal incubations, the contribution of this intestinal metabolism to the elimination of methadone in vivo seems to be relatively small compared to the contribution of hepatic metabolism (Ke et al. 2014). Given that only minor methadone depletion was observed in the incubations with pooled intestinal microsomes (see “In vitro microsomal incubations”), intestinal metabolism was not considered in the model. Conversion of EDDP into EMDP was not included in the model since no EMDP measured in the microsomal incubations with EDDP (see “In vitro microsomal incubations”). The in vitro *V*_max_ obtained from human liver microsomal incubations were scaled to the in vivo situation taking the total liver microsomal protein yield of 32 mg microsomal protein/g liver into account (Barter et al. 2007).

After oral dosing, the urinary excretion of methadone and its metabolites accounts for up to 50% of the given dose (Ånggård et al. 1975; Lugo et al. 2005; Sullivan and Due 1973) with the ratio of unchanged methadone to EDDP ranging between 1/1 and 1/5 (Kharasch et al. 2004, 2009; Verebely et al. 1975). Therefore, urinary excretion of methadone and EDDP was included in the PBK model. In addition, biliary excretion was included in the submodel of EDDP since the recovery of EDDP in feces was reported to account for up to 39% (Foster 2001). The renal clearance of methadone (RCLmet) was set at 1.45 l/h which was the average of the values reported in different in vivo studies (Boulton et al. 2001; Foster et al. 2000; Kharasch et al. 2009). The renal clearance (RCLeddp) and biliary excretion rate constant (kbile) of EDDP were obtained by the curve fitting option in Berkeley Madonna (version 8.3.18, UC Berkeley, CA, USA) in which the steady-state blood maximum concentration (*C*_max_) of EDDP obtained with the PBK model was fitted to the steady-state blood *C*_max_ of EDDP that was reported in subjects receiving methadone maintenance treatment with an oral dose of 57.5 mg/day (De Vos et al. 1995). This resulted in the fitted rate constants for RCLeddp and Kbile of 19.99 l/h and 1.65/h (Table 1), respectively. Kinetic model calculations and curve fitting were performed with Berkeley Madonna, applying Rosenbrock’s algorithms for solving stiff systems. Model equations are shown in supplementary materials 2. Human physiological parameters used in the PBK model were obtained from Brown et al. (1997) (Table 1). Table 2 shows the physicochemical parameters of methadone and EDDP.

**Table 1:** Physiological and biochemical parameters used in the PBK model for methadone and EDDP

Parameters	Symbol	Value	References
Body weight (kg)	BW	70	Brown et al. (1997)
Tissue volume (% body weight)			Brown et al. (1997)
Liver	VLc	0.0257	
Fat	VFc	0.2142	
Lung	VLuc	0.0076	
Arterial blood	VAc	0.0198	
Venous blood	VVc	0.0593	
Kidney	VKc	0.004	
Heart	VHc	0.0047	
Slowly perfused tissue	VSc	0.5318	
Rapidly perfused tissue	VRc	0.052	
Cardiac output (l/h)	Qc	347.9	
Blood flow to tissue (% cardiac output)			Brown et al. (1997)
Liver	QLc	0.227	
Fat	QFc	0.052	
Kidney	QKc	0.175	
Heart	QHc	0.04	
Slowly perfused tissue	QSc	0.188	
Rapidly perfused tissue	QRc	0.318	
Absorption rate constant of methadone (/h)	ka	0.59	Foster et al. (2000); Wolff et al. (2000)
Fraction absorbed of methadone	Fa	0.88	Ke et al. (2014)
Renal clearance of methadone (l/h)	RCLmet	1.45	Boulton et al. (2001); Foster et al. (2000); Kharasch et al. (2009)
Renal clearance of EDDP (l/h)	RCLeddp	19.99^a^	Fitted values
Biliary excretion rate constant of EDDP (/h)	kbile	1.65^a^	Fitted values

^a^Fitted value generated from EDDP data presented in the study of De Vos et al. (1995)

**Table 2:** Physicochemical parameters for methadone and EDDP

Compound	LogP	pKa	BPr	Tissue: blood partition coefficients^d^						
				Liver	Fat	Slowly perfused tissue	Rapidly perfused tissue	Lung	Kidney	Heart
Methadone	3.93^a^	9.20^b^	0.70^c^	12.45	0.46	7.67	12.45	1.77	7.56	4.9
EDDP	4.63^e^	9.64^e^	0.87^c^	11.51	0.18	7.06	11.51	1.56	6.95	4.48

*BPr* blood/plasma ratio

^a^Reported in Ke et al. (2014)

^b^Reported in Gerber et al. (2001)

^c^Reported in Hsu et al. (2013)

^d^Obtained by dividing tissue: plasma partition coefficients by the corresponding BPr values

^e^Obtained from Marvinsketch (ChemAxon)

### Evaluation of the PBK model

To evaluate the performance of the PBK model developed, comparisons were made between predicted blood concentrations and area under the curve (AUC) values of methadone and in vivo blood concentrations and AUC values obtained in clinical studies with repeated daily oral administration at different doses of methadone. Given that the kinetics of methadone were reported based on plasma concentrations in clinical studies, the plasma concentration–time curves were extracted from graphs presented in the respective clinical studies using GetData Graph Digitizer 2.26^1^ and further converted to blood concentration–time curves by multiplying with the BPr value. For the evaluation of the PBK model, the model parameter body weight and the oral dose were chosen to match the values used in the clinical studies. The specifications of in vivo kinetic studies of methadone used to evaluate the PBK model are summarized in Table 4.

### Sensitivity analysis

A local parameter sensitivity analysis was performed to identify influential parameters on the predicted *C*_max_ in the heart venous blood during the steady-state phase. The normalized sensitivity coefficient (SC) was calculated with the following Eq. (4):

4 $${\text{SC}} = \frac{{\left( {C^{\prime} - C} \right)}}{{\left( {P^{\prime} - P} \right)}} \times \frac{P}{C}$$

where *C* is the initial value of the model output being the steady-state *C*_max_ of the heart venous blood, *C*′ is the model output after a 1% increase in each model parameter value, *P* is the initial parameter value and *P*′ is the parameter value after a 1% increase. Parameters with an absolute SC > 0.1 are considered to be influential on the model output (Chiu et al. 2007; Rietjens et al. 2011). The sensitivity analysis was carried out for a subject with a body weight of 70 kg (Brown et al. 1997) and for oral daily doses of 20 and 200 mg, representing, respectively, a clinically relevant dose level and a dose level associated with a high proportion of case reports of cardiotoxicity in subjects receiving methadone (Chou et al. 2014).

### Translation of in vitro concentration–response data to in vivo dose–response data using PBK modeling-based reverse dosimetry

A change in the FPDc in the vitro field potential waveforms can be considered the surrogate endpoint for the QTc interval in the human ECG (Zwartsen et al. 2019). Based on this consideration, PBK modeling-based reverse dosimetry was applied to translate in vitro concentration–response data on FPDc obtained from the hiPSC-CM using the MEA to in vivo dose–response curves for QTc. To this purpose, the in vitro unbound concentrations of methadone tested in the hiPSC-CM MEA assay were set equal to the unbound steady-state *C*_max_ of methadone in the heart venous blood by correcting the fraction unbound in plasma to a fraction unbound in blood using the BPr value in Eq. (5):

5 $$C_{{\text{total, in vitro}}} \cdot f_{{\text{u, m}}} = C_{{\text{total, human blood}}} \cdot \frac{{f_{{\text{u, p}}} }}{{BP_{{\text{r}}} }}$$

where ${C}_{\mathrm{t}\mathrm{o}\mathrm{t}\mathrm{a}\mathrm{l}, \mathrm{i}\mathrm{n} \mathrm{v}\mathrm{i}\mathrm{t}\mathrm{r}\mathrm{o}}$ and ${f}_{\mathrm{u}, \mathrm{m}}$ are the in vitro methadone concentration and unbound fraction of methadone in the in vitro exposure medium, respectively. BPr is the blood to plasma ratio of methadone and ${f}_{\mathrm{u}, \mathrm{p}}$ is the unbound fraction of methadone in human plasma. ${C}_{\mathrm{t}\mathrm{o}\mathrm{t}\mathrm{a}\mathrm{l}, \mathrm{h}\mathrm{u}\mathrm{m}\mathrm{a}\mathrm{n} \mathrm{b}\mathrm{l}\mathrm{o}\mathrm{o}\mathrm{d}}$ values were extrapolated to in vivo oral doses by PBK modeling-based reverse dosimetry, using a bodyweight of 70 kg (Brown et al. 1997). The same procedure was performed for each of the in vitro concentrations tested in the MEA. Thus, the entire in vitro concentration–response curve was translated to a predicted in vivo dose–response curve.

### Evaluation of the PBK modeling-based reverse dosimetry approach

To evaluate the performance of the PBK modeling-based reverse dosimetry approach, the predicted dose–response curves were compared to dose–response data for QTc prolongation obtained from published literature including single case reports, case series (Table S1), cross-sectional, retrospective and prospective studies (Table S2). To better illustrate the dose-dependent effect of methadone on QTc prolongation, individuals who have potential QTc prolonging risk factors including structural heart disease, electrolyte imbalance, hepatic impairment, concomitant use of medications that potentially prolong QTc or influence the metabolism of methadone (Stringer et al. 2009) were excluded from case reports and case series used for the evaluation. Similar criteria could not be applied to the cross-sectional, retrospective and prospective studies due to the absence of detailed individual information on these risk factors. Potential QTc prolonging risk factors and exclusion criteria for these studies are summarized in Table S2. To facilitate the comparison between in vitro- and in vivo-derived values, both the absolute FPDc values obtained from the in vitro cardiotoxicity assay and the in vivo methadone-induced QTc prolongation on ECG were expressed as relative percentages by dividing the post-treatment FPDc and QTc values by the respective baseline values. For the studies in which baseline QTc data were not reported, a population baseline QTc was assumed as described in the study of Florian et al. (2012) in which baseline QTc was set equal to baseline QTc values identified in Wedam et al. (2007), with an average value of 407 ms (411 ms for female; 405 ms for male).

### Benchmark dose modeling

BMD analysis of predicted in vivo dose–response curves was performed to derive a BMD that can be used as point of comparison to evaluate the predicted dose–response data against therapeutic methadone levels reported in the literature. The benchmark response (BMR) was defined as a 10% change compared to the control. For the QTc, an effect of 10% change over the population baseline of 407 ms, amounting to a QTc of 450 ms is frequently used as a threshold for abnormal QTc prolongation (Anchersen et al. 2009; Chou et al. 2014; ICH 2005b; Mujtaba et al. 2013; Treece et al. 2018). The BMD values resulting in a BMR of 10% with lower and upper 95% confidence limit were defined as BMDL_10_ and BMDU_10_. The European Food Safety Authority (EFSA) web-tool^2^ integrated with the R-package PROAST version 66.40 developed by the Dutch National Institute for Public Health and the Environment (RIVM) was used for BMD analysis. In short, the continuous data from the predicted in vivo dose–response curves were fitted to a set of models including the Exponential, Hill, Inverse Exponential, and the Log-Normal Family models. According to the flow-chart described in the manual^2^ provided by EFSA, all fitted models excluding the FULL and NULL model were used for model averaging and a weighted average model was constructed to estimate model averaged confidence intervals using bootstrap sampling (Wheeler and Bailer 2007). Weighting was based on the model’s Akaike's Information Criterion (AIC) values where models with lower AIC values get a larger weight. 200 bootstrap data sets were run to calculate the final BMD confidence intervals from model averaging.

In vitro concentration–response cardiotoxicity data were analyzed using the same BMD approach to derive benchmark concentrations that induced a 20% change in the FPDc over the control (BMC_20_) for comparing the potency of methadone, EDDP and EMDP. The final BMC_20_ values were obtained by weighted averaging BMC_20_ values derived from all fitted models excluding the FULL and NULL model. For this analysis, a BMR of 20% was chosen being the lowest BMR allowing reliable curve fitting.

## Results

### In vitro cardiotoxicity in the hiPSC-CM MEA assay

**Fig. 4:**
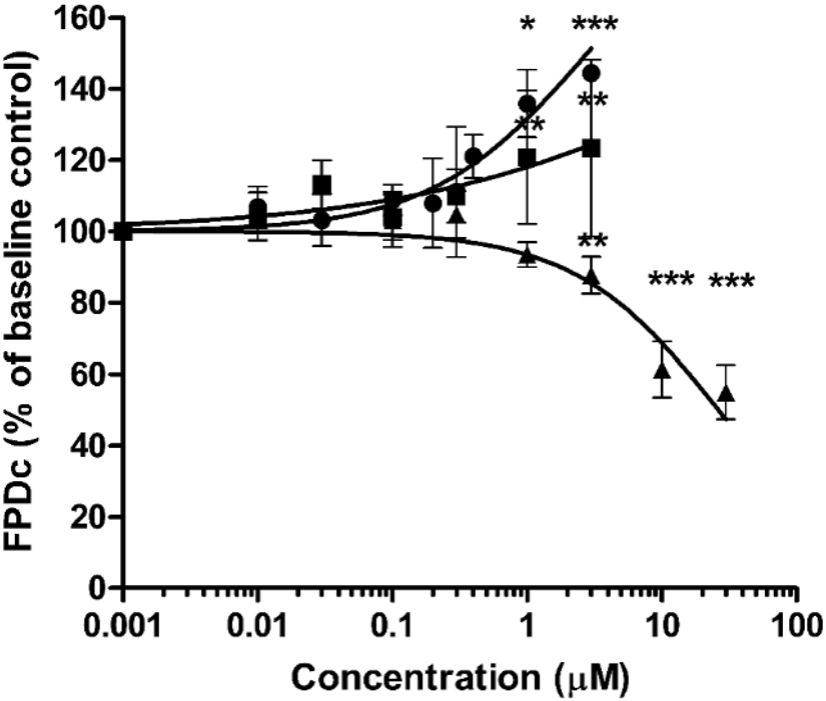
Concentration–response curves for the effect of methadone (circles), EDDP (squares) and EMDP (triangles) on FPDc in hiPSC-CM detected by the MEA. The response of the baseline condition (0.1% (v/v) DMSO) was set at 100%. Data represent the mean of 4–9 wells with in total 26–38 electrodes. Each data point represents the mean ± SD. Statistically significant changes in response compared to the solvent control are marked with * with *p* < 0.05: *, *p* < 0.01: ** and *p* < 0.001: ***

Figure 4 shows the cardiotoxicity of methadone, EDDP and EMDP in hiPSC-CM as detected in the MEA. Methadone and its primary metabolite EDDP significantly prolonged the FPDc in a concentration-dependent manner with a BMC_20_ of 0.6 µM and 2.3 µM, respectively. Of interest to note is that the secondary metabolite EMDP induced an opposite effect, shortening the FPDc in a concentration-dependent manner with the concentration shortening the FPDc by 20% amounting to 3.8 µM. Both methadone and EDDP induced arrhythmia-like waveforms from 3 µM onwards; while, cessation of beating was observed upon the treatment of the hiPSC-CM with methadone and EDDP at 30 µM. EMDP caused beating arrest in certain wells at 30 µM without inducing arrhythmia-type waveforms within the test concentration range. The FPDc of hiPSC-CM treated with repeated application of 0.1% (v/v) DMSO in the vehicle control well was not significantly affected (Fig. S3).

### In vitro microsomal incubations

**Fig. 5:**
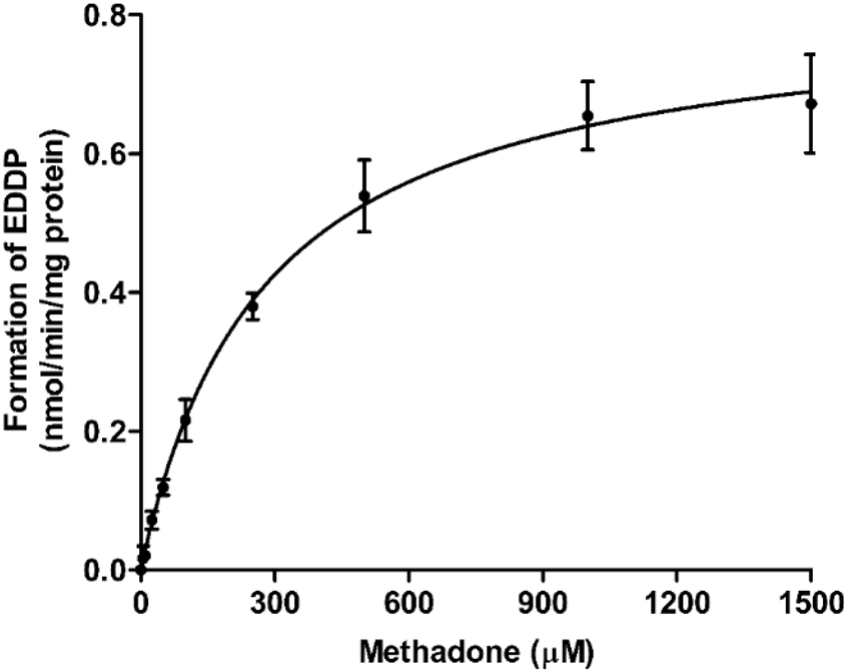
Concentration-dependent formation of EDDP in incubations with human liver microsomes. Data represent the mean of three independent experiments. Each data point represents the mean ± SD

Figure 5 shows the concentration-dependent formation rate of EDDP from methadone by human liver microsomes, which followed Michaelis–Menten kinetics. The apparent *V*_max_ and *K*_m_ values obtained from the data, and the catalytic efficiency *(V*_max_/*K*_m_) are presented in Table 3. No EMDP formation was measured in these incubations. In similar incubations using EDDP as the substrate, formation of EMDP was neither detectable. In incubations with intestinal microsomes applying the two highest methadone concentrations tested in liver microsomes (1000 and 1500 µM), formation of EDDP was less than 8% of the formation observed with liver microsomes at these concentrations. In addition, negligible formation of EDDP was observed in the incubation of 50-µM methadone with increasing incubation time up to 120 min and protein concentrations up to 2-mg/ml human intestinal microsomal protein. Also in these incubations, no EMDP formation was detected. This implied that conversion by intestinal microsomes was considered limited compared to conversion by human liver microsomes and therefore methadone conversion by intestinal tissue was not incorporated in the PBK model and, hence, no further kinetic constants were derived.

**Table 3:** Kinetic constants for formation of EDDP from methadone obtained from in vitro incubations with human liver and intestinal microsomes

Organ	Substrate	Metabolite	*V*_max_ ± SD (nmol/min/mg microsomal protein)	*K*_m_ ± SD (µM)	Catalytic efficiency (µl/min/mg microsomal protein)^a^
Liver	Methadone	EDDP	0.82 ± 0.026	275 ± 26.78	2.97
		EMDP	n.d	n.d	–
	EDDP	EMDP	n.d	n.d	–
Intestine	Methadone	EDDP	0.058, 0.057 ^b^	n.d	–
		EMDP	n.d	n.d	–

*n.d.* not determined, since EDDP and EMDP were unable to be quantified (see text for details)

^a^*V*_max_/*K*_m_ × 1000

^b^Formation rate at 1000 µM and 1500 µM

–Unable to calculate

### Unbound fraction for methadone in in vitro hiPSC-CM MEA medium and in human plasma

Due to the use of serum-free medium in the hiPSC-CM MEA assay, the unbound fraction of methadone in the in vitro medium was relatively high, amounting to 0.79 ± 0.041 compared to the unbound fraction in pooled human plasma determined to be 0.055 ± 0.011. The unbound fraction of EDDP in the in vitro medium was 0.90 ± 0.072 and was 0.30 ± 0.015 in pooled human plasma.

Considering the large inter-individual variation in plasma protein binding for methadone observed in in vivo studies (Eap et al. 1990; Olsen 1973; Romach et al. 1981; Wilkins et al. 1997), also two extreme *f*_u,p_ values (0.034 and 0.22) obtained from the literature together with the Simcyp-derived and RED-derived *f*_u,p_ values (0.15 and 0.055, respectively) were used to translate in vitro effect concentrations to the total blood concentration as presented in Eq. (5), which were subsequently subject to PBK modeling-based reverse dosimetry.

### PBK model development and evaluation

**Fig. 6:**
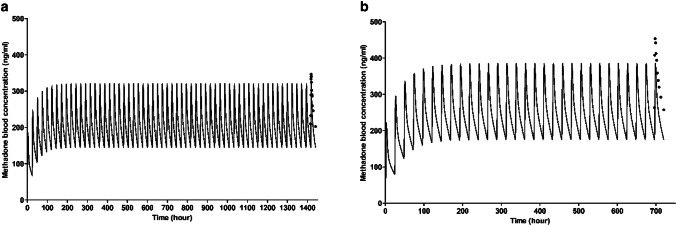
Blood concentration–time curves of methadone in human predicted with the PBK model (lines) and published in vivo data (dots) after a repeated oral dose of 70 mg/day for 60 days **a** (Foster et al. 2000) and 100 mg/day for 30 days **b** (Liu et al. 2007)

To evaluate the performance of the human PBK model, the predicted methadone blood kinetics were compared to in vivo human data obtained from the literature. The specifications of in vivo studies on the subjects receiving methadone maintenance treatment that are used for the PBK model evaluation are summarized in Table 4. As illustrated in Fig. 6, the developed PBK model accurately predicts the change of methadone blood concentrations during the last 24 h upon repeated oral methadone exposure as described in the study of Foster et al. (2000) and Liu et al. (2007). Table 4 further shows the detailed comparison between the model prediction and the in vivo kinetic data using steady-state blood *C*_max_ and AUC values on the last day of exposure as model outcomes. For methadone, the predicted kinetic values are in accordance with reported values expressing a 0.78- to 1.35-fold difference in *C*_max_ values and 0.76- to 0.97-fold difference in AUC values (Table 4).

**Table 4:** Summary of in vivo kinetic studies and evaluation of the PBK model predictions for methadone steady-state blood *C*_max_ and AUC values based on the data derived from in vivo kinetic studies

Mean body weight (kg)	Mean methadone dose (mg/day)^a^	In vivo *C*_max_ (ng/ml) ^b^	In vivo AUC (ng · h/ml) ^b^	Predicted *C*_max_ (ng/ml)	Predicted AUC (ng · h/ml)	Ratio predicted *C*_max_/in vivo C_max_	Ratio predicted AUC/in vivo AUC	References
74	70	346.2	5097	320.5	4967	0.93	0.97	Foster et al. (2000)^c^
90	100	453.6	7889	385.2	5969	0.85	0.76	Liu et al. (2007)^c^
70^d^	61	216.0	n.r	293.1	4542	1.35	–	Diong et al. (2014)
64.7	57.5	383.6	5978	296.4	4591	0.78	0.77	De Vos et al. (1995)

*n.r.* not reported, - unable to calculate

^a^Free base form of methadone

^b^Blood data were obtained by multiplying reported plasma data by the BPr value

^c^In vivo *C*_max_ and AUC is the sum of data of enantiomers

^d^The body weight of subjects was set equal to the value used in the PBK model since body weight of study subjects was not reported

### Sensitivity analysis

**Fig. 7:**
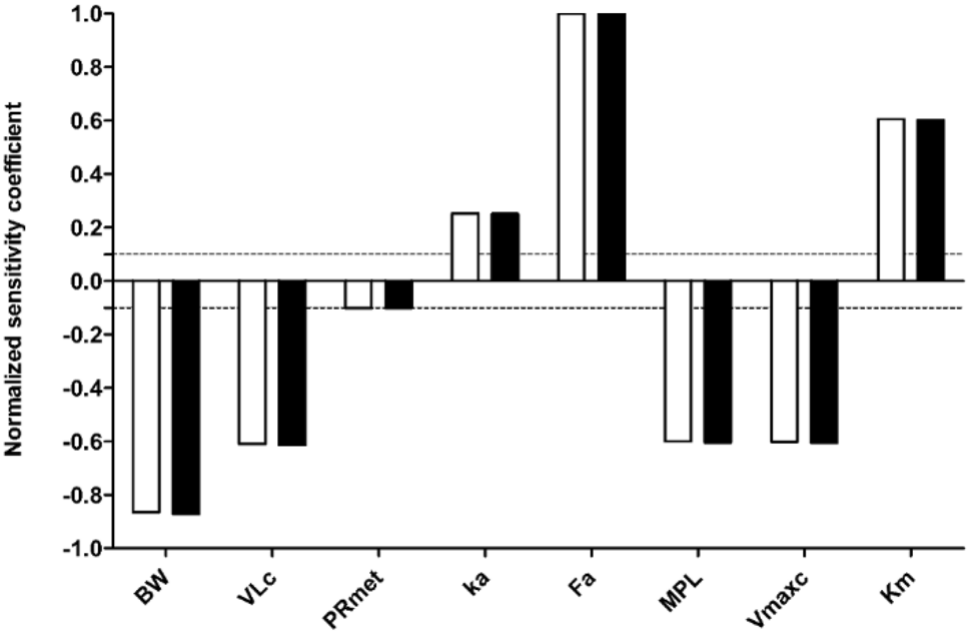
Normalized SCs of PBK model parameters for the prediction of steady-state *C*_max_ of methadone in the heart venous blood upon oral repeated doses of 20 mg/day (white bars) and 200 mg/day (black bars). Model parameters with normalized SC with an absolute value higher than 0.1 (dotted lines) are shown. *BW* body weight, *VLc* fraction of liver, *PRmet* partition coefficient rapidly perfused tissue:blood of methadone, *ka* absorption rate constant, *Fa* oral fraction absorbed, *MPL* liver microsomal protein yield, *Vmaxc* unscaled maximum rate of methadone metabolism in liver, *Km* Michaelis–Menten constant for methadone metabolism in liver

Figure 7 shows the most influential model parameters for the prediction of steady-state *C*_max_ in the heart venous blood upon exposure to oral repeated methadone doses of 20 and 200 mg. The results indicate that the normalized sensitivity coefficients of all PBK model parameters were not dose dependent until at least 200 mg/day and that the predicted steady-state *C*_max_ in the heart venous blood is most sensitive to the oral fraction absorbed and the body weight with normalized SC values above 0.8. The parameters related to liver metabolism (volume of liver, liver microsomal protein yield, unscaled maximum rate of methadone metabolism) also substantially influence the model outcome with normalized SC values of 0.6. The absorption rate constant and the partition coefficient rapidly perfused tissue to blood of methadone are less influential with normalized SC value of 0.25 and 0.1, respectively.

### Translation of in vitro concentration–response data into in vivo dose–response data using PBK modeling-based reverse dosimetry

Although EDDP induced concentration-dependent prolongation of FPDc in the in vitro assay, the free blood *C*_max_ of EDDP, after an oral dose of 57.5 mg/day, was estimated to be 0.05 µM based on EDDP data reported in De Vos et al. (1995). Using the current PBK model, the free blood *C*_max_ of EDDP was predicted to be 0.17 µM at a relatively high dose level of methadone of 200 mg/day. Both the reported and predicted free blood *C*_max_ of EDDP are substantially lower than unbound concentrations causing cardiotoxicity in the hiPSC-CM MEA assay (unbound BMC_20_ = 2.07 µM) (Fig. 4). To reach the unbound BMC_20_ value of 2.07 µM, a methadone dose level of 2600 mg/day was estimated to be required, which is 22-fold higher than the highest clinical relevant dose of 120 mg/day (Chou et al. 2014). Therefore, the cardiotoxicity of EDDP was not considered to play a role in methadone-induced cardiotoxicity and, thus, also not considered for the reverse dosimetry.

**Fig. 8:**
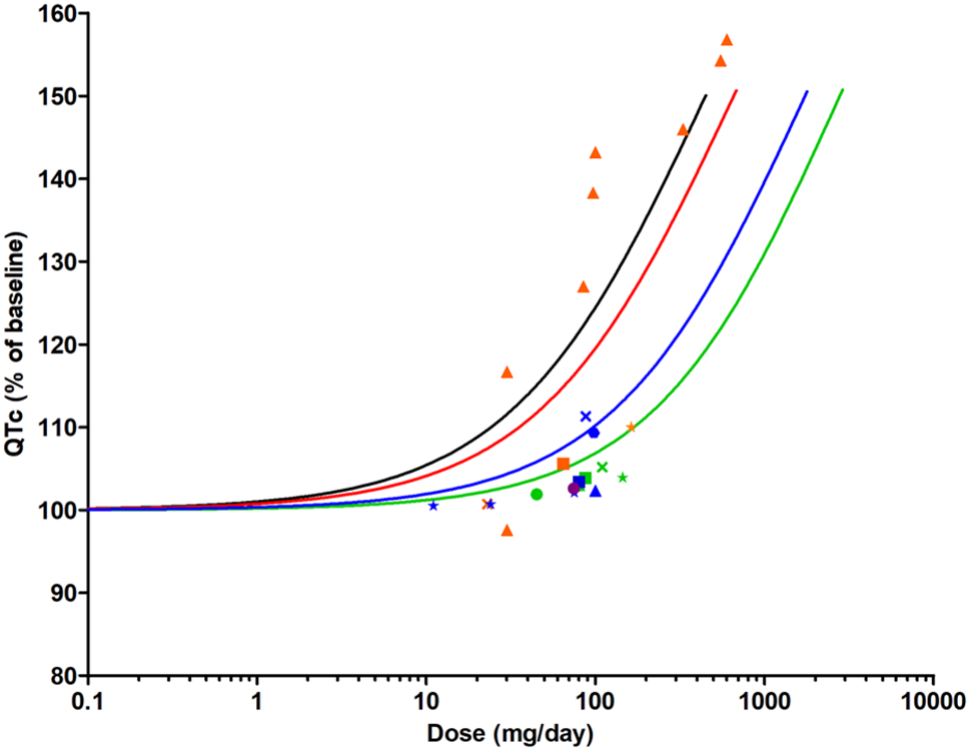
Predicted dose–response curves for cardiotoxicity of methadone obtained using PBK modeling-based reverse dosimetry compared to in vivo dose–response data derived from literature. The curves represent the prediction based on a *f*_u,p_ of 0.22 (black line), 0.15 (red line), 0.055 (blue line) and 0.034 (green line). Symbols represent the data obtained from case reports, case series of individuals (orange triangles) (Esses et al. 2008; Fredheim et al. 2006; Krantz et al. 2002) and other studies as follows: Bart et al. (2017) (purple circle); Carlquist et al. (2015) (orange square); Chang et al. (2012) (green circle) Chowdhury et al. (2015); (dark blue cross); Cruciani et al. (2005) (green cross) Eap et al. (2007); (green star); Ehret et al. (2006) (dark blue triangle) Fareed et al. (2013); (dark blue circle) Heesch et al. (2015); (dark blue star) Krantz et al. (2005); (orange circles) Maremmani et al. (2005); (green square); Martell et al. (2005) (green triangle) Peles et al. (2007); (orange star); Reddy et al. (2010) (orange circles) Roy et al. (2012); (dark blue square). The in vivo data are summarized in Table S1 and S2 (color figure online)

Upon correction for protein binding performed using the values for *f*_u,m_ and *f*_u,p_ described above, the in vitro concentration–response curve of methadone obtained in the hiPSC-CM as detected by the MEA was translated to in vivo dose–response curves for human cardiotoxicity using the developed PBK model. As mentioned in the “unbound fraction for methadone” section, *f*_u.m_ of 0.79 was used to correct for protein binding of methadone in the in vitro medium; while for the in vivo situation, four different *f*_u,p_ values were used including the experimental *f*_u,p_ value obtained from pooled human plasma, an in silico-derived *f*_u,p_ value and two extreme *f*_u,p_ values obtained from the literature (Eap et al. 1990; Foster et al. 2000; Moody et al. 2008; Olsen 1973; Romach et al. 1981; Wilkins et al. 1997). This resulted in four predicted in vivo dose–response curves for methadone-induced cardiotoxicity, one for each of the *f*_u,p_ values (Fig. 8). These predicted dose–response curves were subsequently compared to available in vivo human data.

### Evaluation of the PBK modeling-based reverse dosimetry approach and BMD analysis of predicted dose–response data

To evaluate the performance of the PBK modeling-based reverse dosimetry approach, the dose–response data for QTc prolongation obtained from case reports, case series, cross-sectional, retrospective and prospective studies were compared with the predicted dose–response curves for QTc prolongation taking different *f*_u,p_ values into account. This comparison, presented in Fig. 8, reveals that the predicted in vivo dose–response curves for QTc prolongation were comparable with reported in vivo data. The prediction of QTc prolongation with the *f*_u,p_ value of 0.15 obtained from Simcyp is best in line with the majority of reported QTc prolongation data of individual cases. The QTc prolongation data reported in population studies, however, were more close to the predicted dose–response curve with the *f*_u,p_ value of 0.055 obtained from the RED assay.

**Fig. 9:**
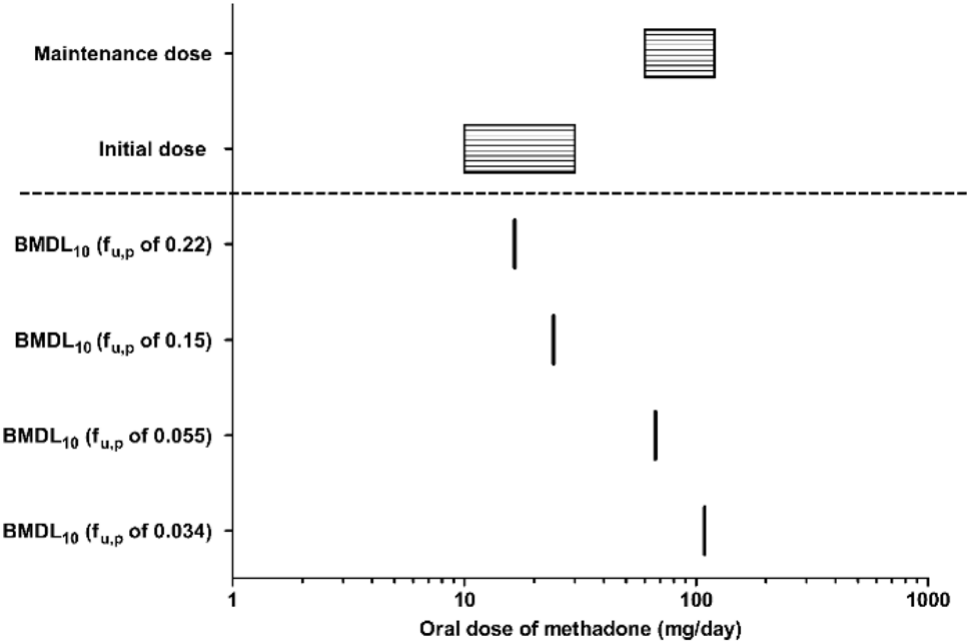
Comparison of BMDL values derived from the predicted dose–response curves for human cardiotoxicity of methadone presented in Fig. 8 (lines) and therapeutic dose levels reported in the literature (boxes filled with horizontal lines)

To further evaluate the model predictions a BMD analysis was performed. BMDL_10_ values were derived and used as points of comparison. Figure 9 presents the BMDL_10_ derived from the dose–response curves presented in Fig. 8, predicted with the different *f*_u,p_ values while also presenting therapeutic dose levels of methadone. The comparison presented in Fig. 9 reveals that the predicted BMDL_10_ values overlap with the therapeutic methadone dose levels. The predicted BMDL_10_ values for methadone-induced cardiotoxicity based on high *f*_u,p_ values of 0.22 and 0.15 are 1.7- and 2.4-fold higher, respectively, than the recommended initial dose for opioid-native patients (10 mg/day), and the predicted BMDL_10_ values based on low *f*_u,p_ values of 0.055 and 0.034 are 2.2- and 3.6-fold higher, respectively, than the recommended initial dose for opioid users (30 mg/day) (Chou et al. 2014; BCCSU 2017). This indicated that these therapeutic dose levels are below the dose levels predicted to result in 10% change, an effect size that can be used as a threshold to evaluate abnormal QTc prolongation (Anchersen et al. 2009; Chou et al. 2014; ICH 2005b; Mujtaba et al. 2013; Treece et al. 2018). The maintenance dose of 60–120 mg methadone/day (Chou et al. 2014; BCCSU 2017) is, however, 0.6- to 7.2-fold higher than the predicted BMDL_10_ values in all scenarios, pointing at a potential cardiotoxic effect in especially individuals with relatively lower plasma protein binding (higher *f*_u,p_). Detailed information on the BMD analysis can be found in the supplementary materials 1 Tables S3–S7 and the BMD values are summarized in Table S7.

## Discussion

The aim of the present study was to investigate whether human in vivo cardiotoxicity could be predicted by a novel testing strategy combining the in vitro toxicity assay with hiPSC-CM in a MEA and PBK modeling-based reverse dosimetry. Methadone was used as the model compound given that for this drug, both kinetic and clinical human data for evaluation of predictions made were available.

The in vitro electrophysical cardiotoxicity was detected using hiPSC-CM combined with the MEA technology, which can capture the overall effects on multiple ion channels on the extracellular field potential. The change in FPDc in the in vitro obtained field potential waveforms can be considered a surrogate endpoint for the QTc interval in the human ECG (Zwartsen et al. 2019), the parameter known to be indicative for methadone-induced cardiotoxicity (Mujtaba et al. 2013). The results show that methadone induced a concentration-dependent prolongation of FPDc which is in line with the study of Kuryshev et al. (2010) reporting that methadone prolonged the action potential duration using patch clamp recordings in human cardiomyocytes. Studies using mammalian cells transfected with cardiac ion channels revealed that the prolonged effect on FPDc can be ascribed to the inhibition of the hERG and sodium channels (Eap et al. 2007; Kuryshev et al. 2010). The major methadone metabolite EDDP appeared to also prolong the FPDc albeit with lower potency than methadone. This lower potency of EDDP is in line with the fact that EDDP was reported to be a weaker hERG channel blocker compared to methadone (Eap et al. 2007; Katchman et al. 2002); while, effects on other ion channels such as sodium channel may contribute to the observed EDDP-induced FPDc prolongation effect (Mishra et al. 2014). Neither FPDc prolongation nor arrhythmia-type waveforms were observed upon exposure of the hiPSC-CM to EMDP which is in accordance with the previous study reporting EMDP to not inhibit hERG channels (Eap et al. 2007). Given that the in vivo total plasma concentration of EMDP has been reported to be less than 0.04 µM after clinical relevant dosing (Alburges et al. 1996), it can be concluded that the in vitro effects of EMDP in the hiPSC-CM MEA assay, with an in vitro BMC_20_ for decreasing FPDc of 3.8 µM, would not be relevant in in vivo. Thus, the methadone-induced prolongation of the FPDs is unlikely to be counteracted by EMDP and the cardiotoxicity of EMDP was not further taken into account.

The evaluation of the developed PBK model against literature data available on steady-state blood *C*_max_ and AUC values of methadone (De Vos et al. 1995; Diong et al. 2014; Foster et al. 2000; Liu et al. 2007), indicated that the model was able to adequately predict the kinetics of methadone with differences being less than twofold, which is generally accepted as an adequate predictive performance (Badhan et al. 2019; WHO 2010).

It is generally assumed that the unbound concentration is responsible for the clinical response of a drug (Smith et al. 2010). Given that methadone is a lipophilic drug with basic properties, the extent of protein binding may play an important role in determining the free concentration and influence the therapeutic or toxic effects of methadone. Given that in vivo experimental data report variation in the *f*_u,p_ values of methadone, the PBK modeling-based reverse dosimetry was performed taking into account different values for *f*_u,p_. It is reported that the fraction of unbound methadone is significantly correlated to the plasma concentration of alpha1-acid glycoprotein (AAG) (Abramson 1982; Yang et al. 2006), which is known to be influenced by physiological and pathologic conditions of the subject (Eap et al. 1990). The 1.5- to 6.5-fold difference in the four f_u,p_ values used for the PBK modeling-based reverse dosimetry are in line with the 2- to 20-fold variation of the AAG concentration among individuals (Taguchi et al. 2013).

The predicted dose–response curves obtained from the PBK modeling-based reverse dosimetry, using the respective *f*_u,p_ values were in line with in vivo data available from case reports, case series, cross-sectional, retrospective and prospective studies on methadone induced effects on in vivo QTc prolongation available in the literature (Fig. 8). This further validates the developed PBK model and provides support for the novel in vitro–in silico testing strategy for prediction of cardiotoxicity in human.

It is of interest to note that the predictions with high *f*_u,p_ values (0.22 and 0.15) are more in line with data obtained from individual case series; while, the data obtained with lower *f*_u,p_ values (0.055 and 0.034) especially match the data from population studies. The reasons underlying this observation remain to be elucidated but may be related to the fact that the concentration of AAG increases under the conditions of heroin addiction (Garrido et al. 2000), HIV infection (Barrail-Tran et al. 2010), and cancer (Huang and Ung 2013). Given that the subjects in the epidemiological studies were associated with those physiological and pathologic conditions, smaller *f*_u,p_ values would be expected; while, the individual case series were selected using criteria that specifically exclude these potential factors that interfere with the concentration of AAG.

To further evaluate the in vitro–in silico predictions for human cardiotoxicity of methadone, BMDL_10_ values derived from predicted dose–response curves were compared to therapeutic doses. The BMDL_10_ values appeared to overlap with the therapeutic dose levels. Given the fact that a BMDL_10_ value is generally considered a dose level that is comparable to a no observed adverse effect level (EFSA 2017), and 10% effect is an effect size used as a threshold to evaluate abnormal QTc prolongation (Anchersen et al. 2009; Chou et al. 2014; ICH 2005b; Mujtaba et al. 2013; Treece et al. 2018), doses lower than the predicted BMDL_10_ values would be expected to be without an effect on QTc prolongation, which is in line with the observation that the predicted BMDL_10_ values based on high *f*_u,p_ values and low *f*_u,p_ values are two- to threefold higher than the recommended initial dose for opioid-native patients (10 mg/day) and opioid users (30 mg/day), respectively (Chou et al. 2014; BCCSU 2017). The fact that the BMDL_10_ values obtained with the relatively higher *f*_u,p_ values are 2.5- to sevenfold lower than the maintenance dose (60 mg/day), may explain the QTc prolongation observed in some methadone maintenance treatment patients given these therapeutic maintenance dose levels. This confirms the need for particular cautions (intensive ECG monitoring and determining arrhythmia risk factor) for patients receiving high doses of methadone (> 100 mg) (Florian et al. 2012; Mujtaba et al. 2013; Treece et al. 2018). Krantz et al. (2002) reported that methadone induced Tdp in patients, without the presence of other risk factors, prescribed an average dose of 400 mg/day, which is consistent with our predictions given the fact that this dose is even fourfold higher than the BMDL_10_ value (109 mg/day) derived from the predictions based on the lowest *f*_u,p_ values. The results of our study indicate that especially subjects with lower levels of plasma protein binding (higher *f*_u,p_) of methadone may be a group at extra risk.

The results of the present study indicate that *f*_u,p_ may be a key parameter causing interindividual differences in the cardiotoxicity of methadone. The exact magnitude of the effect of changes in protein binding on toxicity, however, is not always straightforward since this is an interplay between the available fraction at the site of action, metabolism and excretion and may, i.e., require detailed information on the fate of a compound within cells/the human body which is often not available. Moreover, the variability in other factors that influence the concentration in the heart venous blood may also cause variation in cardiotoxic effects in individuals. Based on the sensitivity analysis, the *C*_max_ in heart venous blood is also influenced by metabolism-related parameters. A major enzyme involved in the metabolism of methadone to EDDP is CYP2B6, a cytochrome P450 that shows large interindividual variability due to genetic polymorphism (Kharasch 2017). It would be of interest to integrate also this variability in the PBK model-based reverse dosimetry approach and predict its influence on the in vivo effects of methadone. This is a topic beyond the aim of the present study, that is currently under investigation. In addition, given that methadone is the racemic mixture of *R*- and *S*-methadone and the latter enantiomer is mainly responsible for the cardiotoxic effects (Ansermot et al. 2010; Eap et al. 2007; Lin et al. 2009), it would also be of interest to predict methadone induced cardiotoxicity distinguishing between the *R*- and *S*-enantiomers.

In the present study, we demonstrated the integration of the hiPSC-CM MEA data and PBK modeling-based reverse dosimetry to assess the in vivo cardiotoxicity of methadone in human. This in vitro–in silico approach enabled the translation of the in vitro concentration–response data on cardiotoxicity to predicted in vivo dose–response data for methadone-induced QTc prolongation in human. Comparison of model predictions to in vivo data revealed that the novel testing strategy provided adequate predictions for both in vivo kinetics and cardiotoxicity of methadone, also pinpointing to an important role for binding to plasma proteins in determining potential interindividual differences in sensitivity towards the cardiotoxic effects of methadone. The present study provides a proof-of-principle of using PBK modeling-based reverse dosimetry for QIVIVE to predict cardiotoxicity in human, providing a novel testing strategy for cardiac safety.

## Electronic supplementary material

Below is the link to the electronic supplementary material.

Supplementary file1 (DOCX 987 kb)

## Abbreviations

ADME: Absorption, distribution, metabolism and excretion
AIC: Akaike’s Information Criterion
AUC: Area under the curve
BMD: Benchmark dose
BMDL_10_—BMDU_10_: Lower and upper 95% confidence limit of BMD resulting in 10% effect
BMR: Benchmark response
BMC_20_: Benchmark concentration that induced a 20% change
EDDP: 2-Ethylidene-1,5-dimethyl-3,3-diphenylpyrrolidine
EMDP: 2-Ethyl-5-methyl-3,3-diphenylpyrroline
FPD: Field potential duration
FPDc: Field potential duration corrected for beat rate
hERG: Human ether-à-go-go-related gene
hiPSC-CM: Human induced pluripotent stem cell-derived cardiomyocytes
C_max_: Maximum concentration
MEA: Multi electrode array
QIVIVE: Quantitative in vitro to in vivo extrapolation
Tdp: Torsade de pointes
*f*_u,p_: Unbound fraction in human plasma
*f*_u,m_: Unbound fraction in in vitro medium

## Footnotes

1 Available at: https://getdata-graph-digitizer.com [Accessed 20th November 2019].

2 EFSA Statistical Models-BMD. [Online]. Available at: https://shiny-efsa.openanalytics.eu/app/bmd [Accessed 20 December, 2019]

## References

[CR1] Abdullah R, Alhusainy W, Woutersen J, Rietjens IMCM, Punt A (2016). Predicting points of departure for risk assessment based on in vitro cytotoxicity data and physiologically based kinetic (PBK) modeling: the case of kidney toxicity induced by aristolochic acid I. Food Chem Toxicol.

[CR2] Abramson FP (1982). Methadone plasma protein binding: alterations in cancer and displacement from α1-acid glycoprotein. Clin Pharmacol Ther.

[CR4] Alburges ME, Huang W, Foltz RL, Moody DE (1996). Determination of methadone and its *N*-demethylation metabolites in biological specimens by GC-PICI-MS. J Anal Toxicol.

[CR5] Alinejad S, Kazemi T, Zamani N, Hoffman RS, Mehrpour O (2015). A systematic review of the cardiotoxicity of methadone. EXCLI J.

[CR6] Anchersen K, Clausen T, Gossop M, Hansteen V, Waal H (2009). Prevalence and clinical relevance of corrected QT interval prolongation during methadone and buprenorphine treatment: a mortality assessment study. Addiction.

[CR7] Ando H (2017). A new paradigm for drug-induced torsadogenic risk assessment using human iPS cell-derived cardiomyocytes. J Pharmacol Toxicol Methods.

[CR8] Ånggård E, Gunne L-M, Holmstrand J, McMahon RE, Sandberg C-G, Sullivan HR (1975). Disposition of methadone in methadone maintenance. Clin Pharmacol Ther.

[CR9] Ansermot N (2010). Substitution of (*R*, *S*)-methadone by (*R*)-methadone: impact on QTc interval. Arch Intern Med.

[CR10] Asakura K (2015). Improvement of acquisition and analysis methods in multi-electrode array experiments with iPS cell-derived cardiomyocytes. J Pharmacol Toxicol Methods.

[CR11] Badhan RK, Gittins R, Al Zabit D (2019). The optimization of methadone dosing whilst treating with rifampicin: a pharmacokinetic modeling study. Drug Alcohol Depend.

[CR12] Barrail-Tran A (2010). Influence of alpha-1 glycoprotein acid concentrations and variants on atazanavir pharmacokinetics in HIV-infected patients included in the ANRS 107 trial. Antimicrob Agents Chemother.

[CR13] Barter ZE (2007). Scaling factors for the extrapolation of in vivo metabolic drug clearance from in vitro data: reaching a consensus on values of human micro-somal protein and hepatocellularity per gram of liver. Curr Drug Metab.

[CR14] Bart G, Wyman Z, Wang Q, Hodges JS, Karim R, Bart BA (2017). Methadone and the QTc interval: paucity of clinically significant factors in a retrospective cohort. J Addict Med.

[CR15] Bell SM (2018). vitro to in vivo extrapolation for high throughput prioritization and decision making. Toxicol Vitro.

[CR16] Berezhkovskiy LM (2004). Determination of volume of distribution at steady state with complete consideration of the kinetics of protein and tissue binding in linear pharmacokinetics. J Pharm Sci.

[CR17] Bernauer U, Oberemm A, Madle S, Gundert-Remy U (2005). The use of in vitro data in risk assessment. Basic Clin Pharmacol Toxicol.

[CR18] Blaauboer BJ (2010). Biokinetic modeling and in vitro–in vivo extrapolations. J Toxicol Environ Health Part B.

[CR19] Boulton DW, Arnaud P, DeVane CL (2001). Pharmacokinetics and pharmacodynamics of methadone enantiomers after a single oral dose of racemate. Clin Pharmacol Ther.

[CR20] British Columbia Centre on Substance Use (BCCSU) (2017) A guideline for the clinical management of opioid use disorder. https://www.bccsu.ca/wp-content/uploads/2017/06/BC-OUD-Guidelines_June2017.pdf. Accessed 20 Nov 2019

[CR21] Brown RP, Delp MD, Lindstedt SL, Rhomberg LR, Beliles RP (1997). Physiological parameter values for physiologically based pharmacokinetic models. Toxicol Ind Health.

[CR22] Carlquist JF (2015). A possible mechanistic link between the CYP2C19 genotype, the methadone metabolite ethylidene-1,5-dimethyl-3,3-diphenylpyrrolidene (EDDP), and methadone-induced corrected QT interval prolongation in a pilot study. Mol Diagn Ther.

[CR23] Chang KC (2012). Gender-specific differences in susceptibility to low-dose methadone-associated QTc prolongation in patients with heroin dependence. J Cardiovasc Electrophysiol.

[CR24] Chiu WA (2007). Evaluation of physiologically based pharmacokinetic models for use in risk assessment. J Appl Toxicol.

[CR25] Chou R (2014). Methadone safety: a clinical practice guideline from the American Pain Society and College on Problems of Drug Dependence, in collaboration with the Heart Rhythm Society. J Pain.

[CR26] Chowdhury M, Wong J, Cheng A, Khilkin M, Palma E (2015). Methadone therapy in underserved urban community: QT c Prolongation and life-threatening ventricular arrhythmias. Cardiovasc Ther.

[CR27] Clements M, Millar V, Williams AS, Kalinka S (2015). Bridging functional and structural cardiotoxicity assays using human embryonic stem cell-derived cardiomyocytes for a more comprehensive risk assessment. Toxicol Sci.

[CR28] Cruciani RA (2005). Measurement of QTc in patients receiving chronic methadone therapy. J Pain Symptom Manag.

[CR29] De Vos J, Ufkes J, van Wilgenburg H, Geerlings P, van den Brink W (1995). Pharmacokinetics of methadone and its primary metabolite in 20 opiate addicts. Eur J Clin Pharmacol.

[CR30] Diong SH (2014). Quantitation of methadone and metabolite in patients under maintenance treatment. J Anal Toxicol.

[CR31] Eap CB (2007). Stereoselective block of hERG channel by (*S*)-methadone and QT interval prolongation in CYP2B6 slow metabolizers. Clin Pharmacol Ther.

[CR32] Eap CB, Cuendet C, Baumann P (1990). Binding of *d*-methadone, 1-methadone, and dl-methadone to proteins in plasma of healthy volunteers: role of the variants of α1-acid glycoprotein. Clin Pharmacol Ther.

[CR33] Eap CB, Buclin T, Baumann P (2002). Interindividual variability of the clinical pharmacokinetics of methadone. Clin Pharmacokinet.

[CR34] Ehret GB (2006). Drug-induced long QT syndrome in injection drug users receiving methadone: high frequency in hospitalized patients and risk factors. Arch Intern Med.

[CR35] Esses JL, Rosman J, Do LT, Schweitzer P, Hanon S (2008). Successful transition to buprenorphine in a patient with methadone-induced torsades de pointes. J Interv Cardiac Electrophysiol.

[CR36] European Food Safety Authority (EFSA) (2017). Update: use of the benchmark dose approach in risk assessment. EFSA J.

[CR37] Ewart L (2012). How do the top 12 pharmaceutical companies operate safety pharmacology?. J Pharmacol Toxicol Methods.

[CR38] Ewart L (2014). The concordance between nonclinical and phase I clinical cardiovascular assessment from a cross-company data sharing initiative. Toxicol Sci.

[CR39] Fareed A, Vayalapalli S, Scheinberg K, Gale R, Casarella J, Drexler K (2013). QTc interval prolongation for patients in methadone maintenance treatment: a five years follow-up study. Am J Drug Alcohol Abuse.

[CR40] Florian J, Garnett C, Nallani S, Rappaport B, Throckmorton D (2012). A modeling and simulation approach to characterize methadone QT prolongation using pooled data from five clinical trials in MMT patients. Clin Pharmacol Ther.

[CR41] Foster DJ (2001) An examination of the metabolism and pharmacokinetics of methadone with respect to stereoselectivity. Dissertation, The Universtiy of Adelaide.

[CR42] Foster DJ, Somogyi AA, Dyer KR, White JM, Bochner F (2000). Steady-state pharmacokinetics of (*R*)-and (*S*)-methadone in methadone maintenance patients. Br J Clin Pharmacol.

[CR43] Foster DJ, Somogyi AA, White JM, Bochner F (2004). Population pharmacokinetics of (*R*)-,(*S*)-and rac-methadone in methadone maintenance patients. Br J Clin Pharmacol.

[CR44] Fredheim OMS, Borchgrevink PC, Hegrenæs L, Kaasa S, Dale O, Klepstad P (2006). Opioid switching from morphine to methadone causes a minor but not clinically significant increase in QTc time: a prospective 9-month follow-up study. J Pain Symptom Manag.

[CR45] Garg P, Garg V, Shrestha R, Sanguinetti MC, Kamp TJ, Wu JC (2018). Human induced pluripotent stem cell–derived cardiomyocytes as models for cardiac channelopathies: a primer for non-electrophysiologists. Circ Res.

[CR46] Garrido M, Aguirre C, Troconiz I, Marot M, Valle M, Zamacona M, Calvo R (2000). Alpha 1-acid glycoprotein (AAG) and serum protein binding of methadone in heroin addicts with abstinence syndrome. Int J Clin Pharmacol Ther.

[CR47] Gerber JG (2001). Effect of ritonavir/saquinavir on stereoselective pharmacokinetics of methadone: results of AIDS clinical trials group (ACTG) 401. J Acquir Immune Defic Syndr (1999).

[CR48] Harris K, Aylott M, Cui Y, Louttit JB, McMahon NC, Sridhar A (2013). Comparison of electrophysiological data from human-induced pluripotent stem cell–derived cardiomyocytes to functional preclinical safety assays. Toxicol Sci.

[CR49] Heesch CB, Copfer AE, Davis SJ, Edwards BW (2015). Evaluation of methadone-induced QTc prolongation in a veteran population. Federal Pract.

[CR50] Hsu Y-C (2013). Methadone concentrations in blood, plasma, and oral fluid determined by isotope-dilution gas chromatography–mass spectrometry. Anal Bioanal Chem.

[CR51] Huang Z, Ung T (2013). Effect of alpha-1-acid glycoprotein binding on pharmacokinetics and pharmacodynamics. Curr Drug Metab.

[CR52] Judson R (2014). In vitro and modelling approaches to risk assessment from the US Environmental Protection Agency ToxCast programme. Basic Clin Pharmacol Toxicol.

[CR53] Justo D, Gal-Oz A, Paran Y, Goldin Y, Zeltser D (2006). Methadone-associated Torsades de Pointes (polymorphic ventricular tachycardia) in opioid-dependent patients. Addiction.

[CR54] Kannankeril P, Roden DM, Darbar D (2010). Drug-induced long QT syndrome. Pharmacol Rev.

[CR55] Katchman AN, McGroary KA, Kilborn MJ, Kornick CA, Manfredi PL, Woosley RL, Ebert SN (2002). Influence of opioid agonists on cardiac humanether-a-go-go-related gene K+ currents. J Pharmacol Exp Ther.

[CR56] Ke AB, Nallani SC, Zhao P, Rostami-Hodjegan A, Unadkat JD (2014). Expansion of a PBPK model to predict disposition in pregnant women of drugs cleared via multiple CYP enzymes, including CYP2B6, CYP2C9 and CYP2C19. Br J Clin Pharmacol.

[CR57] Kharasch ED (2017). Current concepts in methadone metabolism and transport. Clin Pharmacol Drug Dev.

[CR58] Kharasch ED, Hoffer C, Whittington D, Sheffels P (2004). Role of hepatic and intestinal cytochrome P450 3A and 2B6 in the metabolism, disposition, and miotic effects of methadone. Clin Pharmacol Ther.

[CR59] Kharasch ED, Walker A, Whittington D, Hoffer C, Bedynek PS (2009). Methadone metabolism and clearance are induced by nelfinavir despite inhibition of cytochrome P4503A (CYP3A) activity. Drug Alcohol Depend.

[CR60] Kitaguchi T (2017). CSAHi study: detection of drug-induced ion channel/receptor responses, QT prolongation, and arrhythmia using multi-electrode arrays in combination with human induced pluripotent stem cell-derived cardiomyocytes. J Pharmacol Toxicol Methods.

[CR61] Krantz MJ, Lewkowiez L, Hays H, Woodroffe MA, Robertson AD, Mehler PS (2002). Torsade de pointes associated with very-high-dose methadone. Ann Intern Med.

[CR62] Krantz MJ, Lowery CM, Martell BA, Gourevitch MN, Arnsten JH (2005). Effects of methadone on QT-interval dispersion. Pharmacotherapy.

[CR63] Kratz JM, Grienke U, Scheel O, Mann SA, Rollinger JM (2017). Natural products modulating the hERG channel: heartaches and hope. Nat Prod Rep.

[CR64] Kuryshev YA, Kirsch GE, Brown AM (2010). Increased cardiac risk in concomitant methadone and diazepam treatment: pharmacodynamic interactions in cardiac ion channels. Biophys J.

[CR65] Lin C, Somberg T, Molnar J, Somberg J (2009). The effects of chiral isolates of methadone on the cardiac potassium channel IKr. Cardiology.

[CR66] Liu P, Foster G, LaBadie R, Somoza E, Sharma A (2007). Pharmacokinetic interaction between voriconazole and methadone at steady state in patients on methadone therapy. Antimicrob Agents Chemother.

[CR67] Li X, Zhang R, Zhao B, Lossin C, Cao Z (2016). Cardiotoxicity screening: a review of rapid-throughput in vitro approaches. Arch Toxicol.

[CR68] Li H, Zhang M, Vervoort J, Rietjens IMCM, van Ravenzwaay B, Louisse J (2017). Use of physiologically based kinetic modeling-facilitated reverse dosimetry of in vitro toxicity data for prediction of in vivo developmental toxicity of tebuconazole in rats. Toxicol Lett.

[CR69] Louisse J (2010). The use of in vitro toxicity data and physiologically based kinetic modeling to predict dose-response curves for in vivo developmental toxicity of glycol ethers in rat and man. Toxicol Sci.

[CR70] Louisse J, Beekmann K, Rietjens IMCM (2017). Use of physiologically based kinetic modeling-based reverse dosimetry to predict in vivo toxicity from in vitro data. Chem Res Toxicol.

[CR71] Lugo RA, Satterfield KL, Kern SE (2005). Pharmacokinetics of methadone. J Pain Palliat Care Pharmacother.

[CR72] Ma J (2011). High purity human-induced pluripotent stem cell-derived cardiomyocytes: electrophysiological properties of action potentials and ionic currents. Am J Physiol Heart Circ Physiol.

[CR73] Maremmani I, Pacini M, Cesaroni C, Lovrecic M, Perugi G, Tagliamonte A (2005). QTc interval prolongation in patients on long-term methadone maintenance therapy. Eur Addict Res.

[CR74] Martell BA, Arnsten JH, Krantz MJ, Gourevitch MN (2005). Impact of methadone treatment on cardiac repolarization and conduction in opioid users. Am J Cardiol.

[CR75] Martin RL, McDermott JS, Salmen HJ, Palmatier J, Cox BF, Gintant GA (2004). The utility of hERG and repolarization assays in evaluating delayed cardiac repolarization: influence of multi-channel block. J Cardiovasc Pharmacol.

[CR76] Mirams GR (2011). Simulation of multiple ion channel block provides improved early prediction of compounds’ clinical torsadogenic risk. Cardiovasc Res.

[CR77] Mishra H, Polak S, Jamei M, Rostami-Hodjegan A (2014). Interaction between domperidone and ketoconazole: toward prediction of consequent QTc prolongation using purely in vitro information. CPT Pharmacomet Syst Pharmacol.

[CR78] Moody DE, Lin S-N, Chang Y, Lamm L, Greenwald MK, Ahmed MS (2008). An enantiomer-selective liquid chromatography-tandem mass spectrometry method for methadone and EDDP validated for use in human plasma, urine, and liver microsomes. J Anal Toxicol.

[CR79] Mujtaba S, Romero J, Taub CC (2013). Methadone, QTc prolongation and torsades de pointes: current concepts, management and a hidden twist in the tale?. J Cardiovasc Dis Res.

[CR80] Nakamura Y (2014). Assessment of testing methods for drug-induced repolarization delay and arrhythmias in an iPS cell–derived cardiomyocyte sheet: multi-site validation study. J Pharmacol Sci.

[CR81] Nilsson M-I, Meresaar U, ÄNggård E (1982). Clinical pharmacokinetics of methadone. Acta Anaesthesiol Scand.

[CR82] Ning J, Louisse J, Spenkelink B, Wesseling S, Rietjens IMCM (2017). Study on inter-ethnic human differences in bioactivation and detoxification of estragole using physiologically based kinetic modeling. Arch Toxicol.

[CR119] Nozaki Y (2017). CSAHi study-2: validation of multi-electrode array systems (MEA60/2100) for prediction of drug-induced proarrhythmia using human iPS cell-derived cardiomyocytes: assessment of reference compounds and comparison with non-clinical studies and clinical information. Regul Toxicol Pharmacol.

[CR83] Oda Y, Kharasch ED (2001). Metabolism of methadone andlevo-α-acetylmethadol (LAAM) by human intestinal cytochrome P450 3A4 (CYP3A4): potential contribution of intestinal metabolism to presystemic clearance and bioactivation. J Pharmacol Exp Ther.

[CR84] Olsen GD (1973). Methadone binding to human plasma proteins. Clin Pharmacol Ther.

[CR85] Pang L (2019). Workshop report: FDA workshop on improving cardiotoxicity assessment with human-relevant platforms. Circ Res.

[CR86] Peles E, Bodner G, Kreek MJ, Rados V, Adelson M (2007). Corrected-QT intervals as related to methadone dose and serum level in methadone maintenance treatment (MMT) patients—a cross-sectional study. Addiction.

[CR87] Reddy S, Hui D, Osta BE, de la Cruz M, Walker P, Palmer JL, Bruera E (2010). The effect of oral methadone on the QTc interval in advanced cancer patients: a prospective pilot study. J Palliat Med.

[CR88] Redfern W (2003). Relationships between preclinical cardiac electrophysiology, clinical QT interval prolongation and torsade de pointes for a broad range of drugs: evidence for a provisional safety margin in drug development. Cardiovasc Res.

[CR89] Rehnelt S (2017). Frequency-dependent multi-well cardiotoxicity screening enabled by optogenetic stimulation. Int J Mol Sci.

[CR90] Rietjens IMCM, Louisse J, Punt A (2011). Tutorial on physiologically based kinetic modeling in molecular nutrition and food research. Mol Nutr Food Res.

[CR91] Romach M, Piafsky K, Abel J, Khouw V, Sellers E (1981). Methadone binding to orosomucoid (α1-acid glycoprotein): Determinant of free fraction in plasma. Clin Pharmacol Ther.

[CR92] Roy AK, McCarthy C, Kiernan G, McGorrian C, Keenan E, Mahon NG, Sweeney B (2012). Increased incidence of QT interval prolongation in a population receiving lower doses of methadone maintenance therapy. Addiction.

[CR93] Sala L, Ward-van Oostwaard D, Tertoolen LG, Mummery CL, Bellin M (2017). Electrophysiological analysis of human pluripotent stem cell-derived cardiomyocytes (hPSC-CMs) using multi-electrode arrays (MEAs). J Vis.

[CR94] Smith DA, Di L, Kerns EH (2010). The effect of plasma protein binding on in vivo efficacy: misconceptions in drug discovery. Nat Rev Drug Discov.

[CR95] Stevens JL, Baker TK (2009). The future of drug safety testing: expanding the view and narrowing the focus. Drug Discov Today.

[CR96] Strikwold M, Spenkelink B, Woutersen RA, Rietjens IMCM, Punt A (2013). Combining in vitro embryotoxicity data with physiologically based kinetic (PBK) modelling to define in vivo dose–response curves for developmental toxicity of phenol in rat and human. Arch Toxico.

[CR97] Strikwold M, Spenkelink B, de Haan LH, Woutersen RA, Punt A, Rietjens IMCM (2017). Integrating in vitro data and physiologically based kinetic (PBK) modelling to assess the in vivo potential developmental toxicity of a series of phenols. Arch Toxicol.

[CR98] Stringer J, Welsh C, Tommasello A (2009). Methadone-associated QT interval prolongation and torsades de pointes. Am J Health Syst Pharm.

[CR99] Sullivan HR, Due SL (1973). Urinary metabolites of dl-methadone in maintenance subjects. J Med Chem.

[CR100] Taguchi K, Nishi K, Chuang VTG, Maruyama T, Otagiri M, Janciauskiene S (2013). Molecular aspects of human alpha-1 acid glycoprotein—structure and function. Acute phase proteins.

[CR101] The International Council for Harmonisation of Technical Requirements for Pharmaceuticals for Human Use (ICH) (2005a) S7B: The non-clinical evaluation of the potential for delayed ventricular re-polarization (QT interval prolongation) by human pharmaceuticals. https://database.ich.org/sites/default/files/S7B_Guideline.pdf. Accessed 20 Nov 2019

[CR102] The International Council for Harmonisation of Technical Requirements for Pharmaceuticals for Human Use (ICH) (2005b) E14: the clinical evaluation of QT/QTc interval prolongation and proarrhythmic potential for non-antiarrhythmic drugs. https://database.ich.org/sites/default/files/E14_Guideline.pdf. Accessed 20 Nov 2019

[CR103] Thermo Fisher Scientific (2017) User Guide: Single-Use RED Plate with Inserts. https://assets.thermofisher.com/TFS-Assets/LSG/manuals/MAN0011619_SgleUse_RED_Plate_Insert_UG.pdf. Accessed 20 Nov 2019

[CR104] Totah RA, Sheffels P, Roberts T, Whittington D, Thummel K, Kharasch ED (2008). Role of CYP2B6 in stereoselective human methadone metabolism. Anesthesiology.

[CR105] Treece JM (2018). Comprehensive review on methadone-induced QT prolongation and torsades. J Pharmacol Pharmacother.

[CR106] Vandenberk B (2016). Which QT correction formulae to use for QT monitoring?. J Am Heart Assoc.

[CR107] van Liempd S, Morrison D, Sysmans L, Nelis P, Mortishire-Smith R (2011). Development and validation of a higher-throughput equilibrium dialysis assay for plasma protein binding JALA. J Assoc Lab Autom.

[CR108] Verebely K, Volavka J, Mulé S, Resnick R (1975). Methadone in man: pharmacokinetic and excretion studies in acute and chronic treatment. Clin Pharmacol Ther.

[CR109] Wakefield ID, Pollard C, Redfern WS, Hammond TG, Valentin JP (2002). The application of in vitro methods to safety pharmacology. Fundam Clin Pharmacol.

[CR110] Waters NJ, Jones R, Williams G, Sohal B (2008). Validation of a rapid equilibrium dialysis approach for the measurement of plasma protein binding. J Pharm Sci.

[CR111] Wedam EF, Bigelow GE, Johnson RE, Nuzzo PA, Haigney MC (2007). QT-interval effects of methadone, levomethadyl, and buprenorphine in a randomized trial. Arch Intern Med.

[CR112] Wheeler MW, Bailer AJ (2007). Properties of model-averaged BMDLs: a study of model averaging in dichotomous response risk estimation risk analysis. Int J.

[CR113] Wilkins JN, Ashofteh A, Setoda D, Wheatley WS, Huigen H, Ling W (1997). Ultrafiltration using the Amicon MPS-1 for assessing methadone plasma protein binding. Ther Drug Monit.

[CR114] Wolff K, Rostami-Hodjegan A, Hay A, Raistrick D, Tucker G (2000). Population-based pharmacokinetic approach for methadone monitoring of opiate addicts: potential clinical utility. Addiction.

[CR115] World Health Organization (WHO). (2010). Characterization and application of physiologically based pharmacokinetic models in risk assessment. https://www.inchem.org/documents/harmproj/harmproj/harmproj9.pdf. Accessed 20 Nov 2019

[CR116] Yang F, Tong X, McCarver DG, Hines RN, Beard DA (2006). Population-based analysis of methadone distribution and metabolism using an age-dependent physiologically based pharmacokinetic model. J Pharmacokinet Pharmacodyn.

[CR117] Zhao S, Kamelia L, Boonpawa R, Wesseling S, Spenkelink B, Rietjens IMCM (2019). Physiologically based kinetic modeling-facilitated reverse dosimetry to predict in vivo red blood cell acetylcholinesterase inhibition following exposure to chlorpyrifos in the Caucasian and Chinese population. Toxicol Sci.

[CR118] Zwartsen A, de Korte T, Nacken P, de Lange DW, Westerink RH, Hondebrink L (2019). Cardiotoxicity screening of illicit drugs and new psychoactive substances (NPS) in human iPSC-derived cardiomyocytes using microelectrode array (MEA) recordings. J Mol Cell Cardiol.

