# Supplementary material for: Integrating in vitro data and physiologically based kinetic modeling-facilitated reverse dosimetry to predict human cardiotoxicity of methadone
Source: Arch Toxicol. 2020 May 4;94(8):2809–27. doi: 10.1007/s00204-020-02766-7 (PMC7395048; doi:10.1007/s00204-020-02766-7)
Supplement: Supplementary file 1 — Supplementary file1 (DOCX 987 kb) [file 204_2020_2766_MOESM1_ESM.docx]

**Integrating in vitro data and physiologically based kinetic modelling-facilitated reverse dosimetry to predict human cardiotoxicity of methadone**

Miaoying Shi^1^, Hans Bouwmeester^1^, Ivonne MCM Rietjens^1^, Marije Strikwold^2^

^1^ Division of Toxicology, Wageningen University, Stippeneng 4, 6708 WE Wageningen, The Netherlands

^2^ Van Hall Larenstein University of Applied Sciences, 8901 BV Leeuwarden, The Netherlands

*Corresponding author:

Miaoying Shi

Division of Toxicology, Wageningen University

Stippeneng 4, 6708 WE Wageningen, the Netherlands

Tel: +31-317 483334

Fax: +31-317 484931

Email: [miaoying.shi@wur.nl](mailto:miaoying.shi@wur.nl)

**Supplementary materials 1**

**
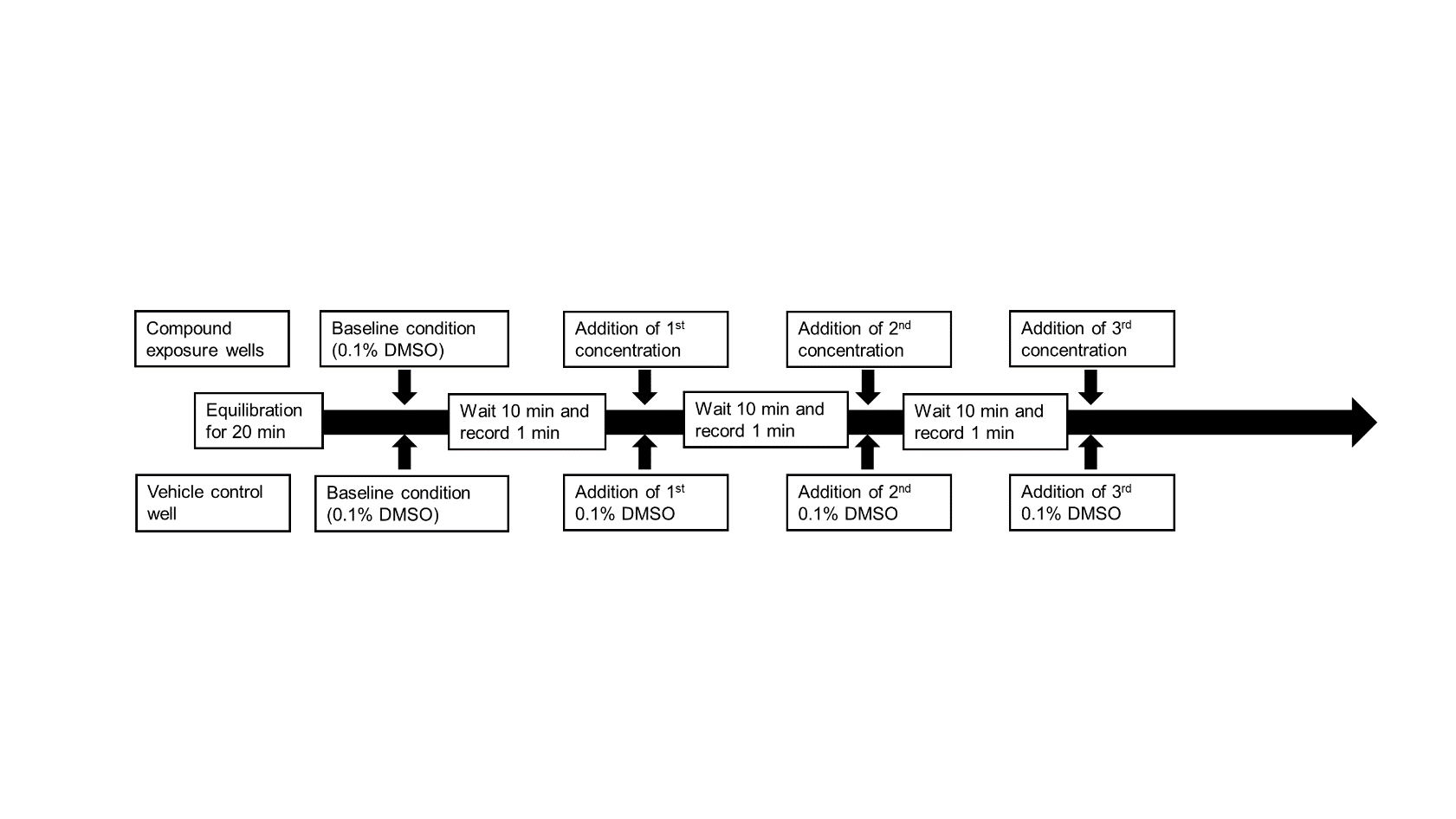
**

**Fig. S1** Schematic time line of the cumulative exposure in the MEA

**
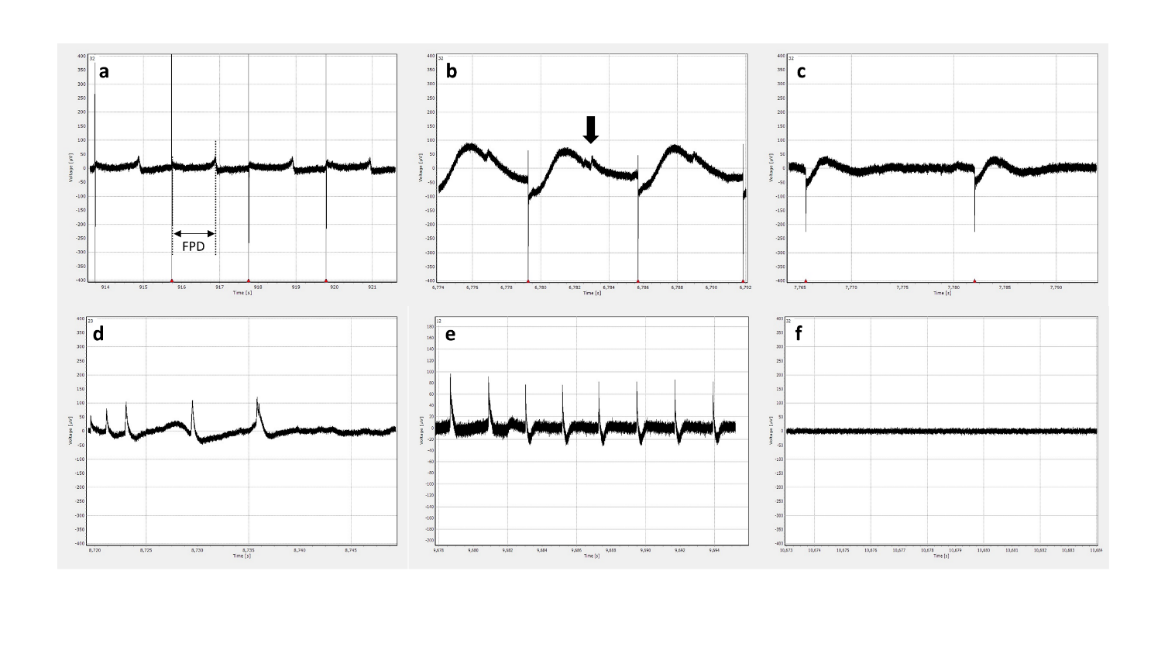
**

**Fig. S2** Different types of waveforms of field potential. a, field potential at baseline condition. b, Arrhythmia-type waveform indicated by the arrow. c-e, waveforms with flattened unclear second peak. f, beating arrest. Waveforms present in b-f were not used for defining the in vitro concentration-response curves for FPDc effects of methadone


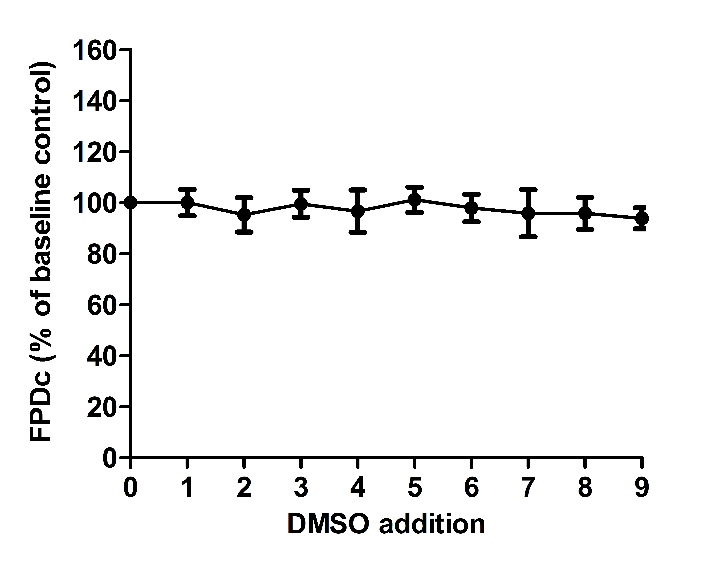


**Fig. S3** Effects of repeated addition of 0.1% (v/v) DMSO on the change of FPDc from baseline condition along time in the vehicle control well. The response of the baseline control (0.1% (v/v) DMSO) was set at 100%, represent as 0 on the X axis in the figure. 1-9 on the X axis represent the 1^st^ to 9^th^ addition of 0.1% (v/v) DMSO corresponding to the 1^st^ to 9^th^ addition of test compounds in the compound exposure wells. Data represent the mean of 3-8 wells with total 17-43 electrodes. Each data point represents the mean ± SD of three independent experiments. Statistical significance was analyzed by one-way ANOVA followed by post Dunnett test in Graph Pad Prism 5.0. No statistically significant changes were found

**Table S1** Summary of clinical case reports of QT prolongation upon use of methadone for opioid addiction or treatment of cancer pain

| Reference ^a^ | Type of study | Age/sex | Dose (mg/day) | Baseline QTc (ms) | Post QTc (ms) | QTc (% to baseline) |
| --- | --- | --- | --- | --- | --- | --- |
| Esses et al. (2008) | case report | 56/male | 100 | 405 ^b^ | 580 | 134.9 |
| Krantz et al. (2002) | case serials | 52/male | 550 | 405 ^b^ | 625 | 145.3 |
|  |  | 60/male | 97 | 405 ^b^ | 560 | 130.2 |
|  |  | 75/female | 330 | 411 ^b^ | 522 | 127.0 |
|  |  | 55/female | 270 | 411 ^b^ | 600 | 133.3 |
|  |  | 47/male | 600 | 405 ^b^ | 635 | 147.7 |
| Fredheim et al. (2006) | individuals from the prospective study | 64/male | 30 | 420 | 410 | 97.6 |
|  |  | 59/male | 30 | 420 | 490 | 116.7 |

^a^ Potential QTc prolonging risk factors including drug-drug interaction, structural heart disease, electrolyte imbalance and hepatic impairment were not present in all cases. ^b^ No baseline information was mentioned in the case study and baseline was assumed to be 407 ms and 411 ms for male and female, respectively (Wedam et al. 2007).

**Table S2** Summary of data from epidemiological studies on adverse effects induced by long term use of methadone

| References | Type of study | Patient population | N (male%) | Dose ± SD mg/day | Duration on methadone | Baseline QTc (ms) | Post-QTc (ms) | QTc % | Presence or absence of potential QTc prolonging risk factors identified in the study | Patient exclusion criteria |
| --- | --- | --- | --- | --- | --- | --- | --- | --- | --- | --- |
| Martell et al. (2005) | prospective | MMT | 160 (66%) | 80 | 6 months | 418.1 ±22 | 430.8±24 | 103.04 | concomitant use of medication (including benzodiazepine); cocaine; alcohol; HCV; HIV; cardiac disease | structural heart disease |
| Krantz et al. (2005) | prospective | MMT | 118 (62%) | 80 ± 32 | 6 months | 415.3 | 429.4 | 103.40 | concomitant use of medications; cocaine; alcohol; | structural heart disease |
| Reddy et al. (2010) | prospective | cancer pain | 64 | 23 | 2 weeks | 427 | 430 | 100.7 | structural heart disease; electrolyte abnormalities; concomitant use of medications | history of arrhythmias, pacemaker or defibrillator; any contraindications to methadone |
| Carlquist et al. (2015) | prospective | MMT | 31 (64.5%) | 64.6 ± 30.5 | 21 days | 389.3 | 411.1 | 105.6 | concomitant use of medications | history of cardiac arrhythmia |
| Chang et al. (2012) | longitudinal | MMT | 150 (84%) | 45 ± 24 | 6 months | 422 ± 24 | 430 ± 23 | 101.9 | no structural heart diseases; no concomitant use of medications | severe physical or mental disorders, cardiac hepatic or renal dysfunction |
| Cruciani et al. (2005) | cross-sectional | MMT and pain | 104 (61%) | 110 | 12.5 months | 407 | 428 | 105.2 | structural heart disease; concomitant use of medications | congenital long QT syndrome; atrial fibrillation; wide QRS complex; |
| Maremmani et al. (2005) | cross-sectional | MMT | 83 (76%) | 87 ± 76 | > 6 months; steady dose for 4 months | 407 | 423 | 103.9 | no other medications known to prolong QTc; normal electrolytes; no morphine, cocaine metabolites, and amphetamines | congenital long QT syndrome; implanted pacemaker; atrial fibrillation or wide QRS complex |
| Peles et al. (2007) | cross-sectional | MMT | 17 | 162.7 ± 28.7 | > 100 days; steady dose > 2 weeks | 407 | 447.6±31.7 | 110.0 | structural heart disease; opiates; cocaine; cannabis; HIV; HCV; HBV | concomitant use of medications |
| Eap et al. (2007) | cross-sectional | MMT | 170 (78%) | 145 ± 83 | 61 ± 61 months | 407 | 423 ±23 | 103.9 | concomitant use of medications (including benzodiazepines); opiate; cocaine; cannabis; alcohol; | n.r. |
| Roy et al. (2012) | cross-sectional | MMT | 180 (61.9%) | 80 ± 27.5 | > 3 months | 407 | 421 | 103.4 | benzodiazepines; opiates; cocaine | concomitant use of medications |
| Ehret et al. (2006) | retrospective | MMT | 167 (66%) | 100 | n.r. | 430 | 440 | 102.33 | concomitant use of medications; HIV; HCV; HBV | severe heart disease; congenital long QT syndrome |
| Fareed et al. (2013) | retrospective | MMT | 32 | 98 ± 55 | 74 ± 31 months | 420 ± 33 | 459 ± 29 | 109.3 | concomitant use of medications; opiates; cocaine; heart diseases | n.r. |
| Heesch et al. (2015) | retrospective | veteran | 45 | 11 | 161 days | 428 | 430 | 100.5 | concomitant use of medications | heart failure; implanted cardiac defibrillate or pacemaker |
|  |  |  | 27 | 24 |  | 436 | 439 | 100.7 |  |  |
|  |  |  | 21 | 75 |  | 437 | 446 | 102.1 |  |  |
| Chowdhury et al. (2015) | retrospective | MMT | 259 | 87.9 | n.r. | 407 | 453 | 111.3 | concomitant use of medications; HIV; heart diseases | n.r. |
| Bart et al. (2017) | retrospective | MMT | 211 (57.4%) | 74.5 ± 26.5 | 1.2 ± 1.6 years | 429 ± 35 | 440 ± 35 | 102.6 | n.r. | atrial fibrillation |

N, number; MMT, methadone maintenance treatment; n.r., not reported.

**Table S3** Results from a BMD analysis of the predicted dose-response curve (f_u,p_ values of 0.22) for human cardiotoxicity of methadone, using PBK modelling-based reverse dosimetry of hiPSC-CM MEA data. The table and figures present the characteristics of fitted models, the weights for model averaging and the final benchmark dose for 10% effect with the 95% lower–upper confidence limit values of the benchmark dose (BMDL_10_-BMDU_10_)

| model | converged | loglik | npar | AIC | weights | **Final BMD values from averaging** | |
| --- | --- | --- | --- | --- | --- | --- | --- |
|  |  |  |  |  |  | **BMDL_10_ (mg/day)** | **BMDU_10_ (mg/day)** |
| full model | yes | 37.87 | 9 | -57.74 | - | **16.5** | **79.5** |
| null model | yes | 22.47 | 2 | -40.94 | - |  |  |
| Expon. m3- | yes | 35.93 | 4 | -63.86 | 0.26 |  |  |
| Expon. m5- | yes | 36.26 | 5 | -62.52 | - |  |  |
| Hill m3- | yes | 35.93 | 4 | -63.86 | 0.26 |  |  |
| Hill m5- | yes | 36.18 | 5 | -62.36 | - |  |  |
| Inv.Expon. m3- | yes | 35.88 | 4 | -63.76 | 0.24 |  |  |
| Inv.Expon. m5- | yes | 36.18 | 5 | -62.36 | - |  |  |
| LN m3- | yes | 35.90 | 4 | -63.80 | 0.24 |  |  |
| LN m5- | yes | 36.20 | 5 | -62.40 | - |  |  |


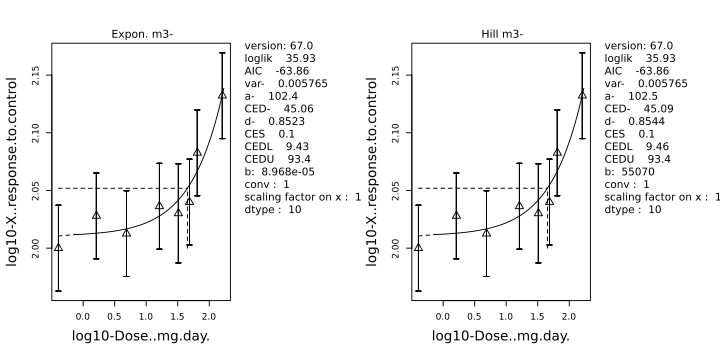

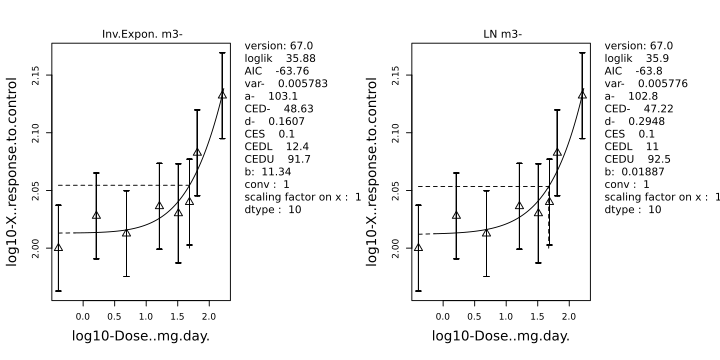


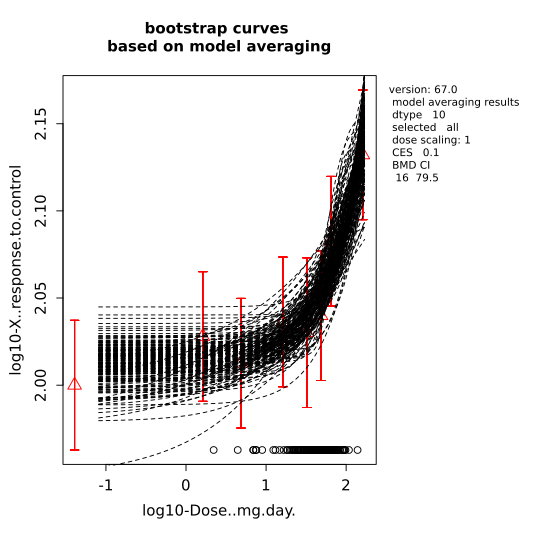


**Table S4** Results from a BMD analysis of the predicted dose-response curve (f_u,p_ values of 0.15) for human cardiotoxicity of methadone, using PBK modelling-based reverse dosimetry of hiPSC-CM MEA data. The table and figures present the characteristics of fitted models, the weights for model averaging and the final benchmark dose for 10% effect with the 95% lower–upper confidence limit values of the benchmark dose (BMDL_10_-BMDU_10_)

| model | converged | loglik | npar | AIC | weights | **Final BMD values from averaging** | |
| --- | --- | --- | --- | --- | --- | --- | --- |
|  |  |  |  |  |  | **BMDL_10_ (mg/day)** | **BMDU_10_ (mg/day)** |
| full model | yes | 37.87 | 9 | -57.74 | - | **24.2** | **117** |
| null model | yes | 22.47 | 2 | -40.94 | - |  |  |
| Expon. m3- | yes | 35.93 | 4 | -63.86 | 0.26 |  |  |
| Expon. m5- | yes | 36.26 | 5 | -62.52 | - |  |  |
| Hill m3- | yes | 35.93 | 4 | -63.86 | 0.26 |  |  |
| Hill m5- | yes | 36.18 | 5 | -62.36 | - |  |  |
| Inv.Expon. m3- | yes | 35.88 | 4 | -63.76 | 0.24 |  |  |
| Inv.Expon. m5- | yes | 36.18 | 5 | -62.36 | - |  |  |
| LN m3- | yes | 35.90 | 4 | -63.80 | 0.24 |  |  |
| LN m5- | yes | 36.20 | 5 | -62.40 | - |  |  |


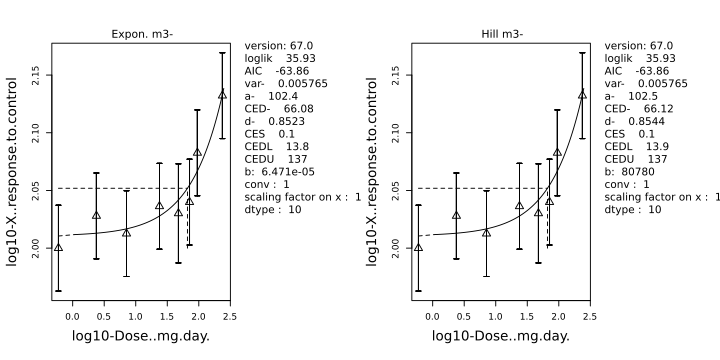

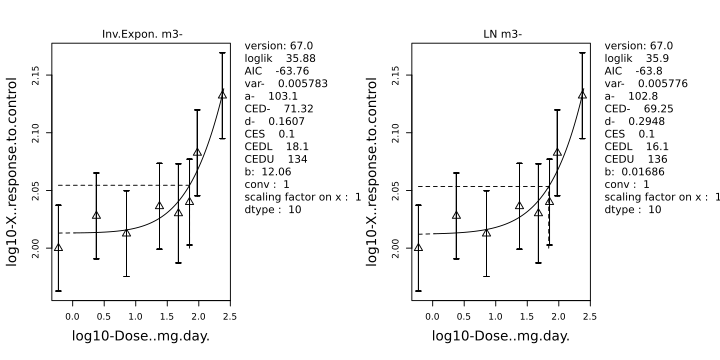


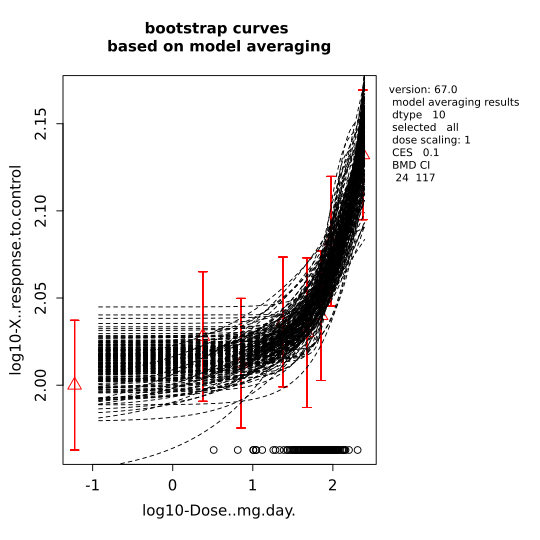


**Table S5** Results from a BMD analysis of the predicted dose-response curve (f_u,p_ values of 0.055) for human cardiotoxicity of methadone, using PBK modelling-based reverse dosimetry of hiPSC-CM MEA data. The table and figures present the characteristics of fitted models, the weights for model averaging and the final benchmark dose for 10% effect with the 95% lower–upper confidence limit values of the benchmark dose (BMDL_10_-BMDU_10_)

| model | converged | loglik | npar | AIC | weights | **Final BMD values from averaging** | |
| --- | --- | --- | --- | --- | --- | --- | --- |
|  |  |  |  |  |  | **BMDL_10_ (mg/day)** | **BMDU_10_ (mg/day)** |
| full model | yes | 37.87 | 9 | -57.74 | - | **66.7** | **316** |
| null model | yes | 22.47 | 2 | -40.94 | - |  |  |
| Expon. m3- | yes | 35.93 | 4 | -63.86 | 0.26 |  |  |
| Expon. m5- | yes | 36.26 | 5 | -62.52 | - |  |  |
| Hill m3- | yes | 35.93 | 4 | -63.86 | 0.26 |  |  |
| Hill m5- | yes | 36.18 | 5 | -62.36 | - |  |  |
| Inv.Expon. m3- | yes | 35.88 | 4 | -63.76 | 0.24 |  |  |
| Inv.Expon. m5- | yes | 36.18 | 5 | -62.36 | - |  |  |
| LN m3- | yes | 35.90 | 4 | -63.80 | 0.24 |  |  |
| LN m5- | yes | 36.20 | 5 | -62.40 | - |  |  |


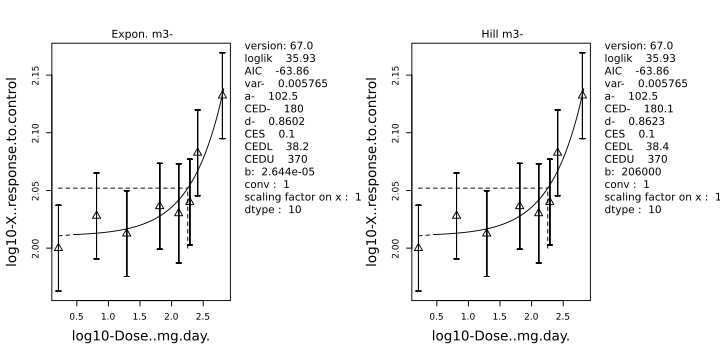

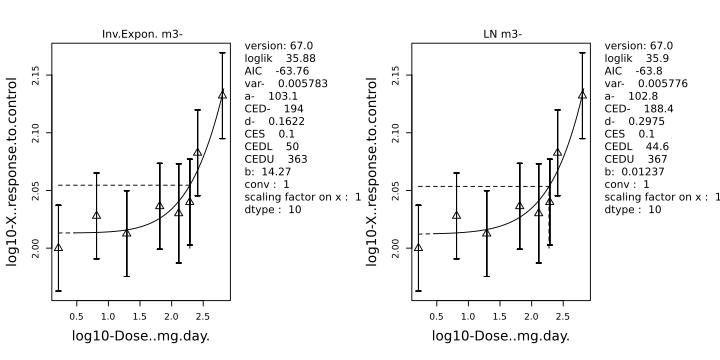

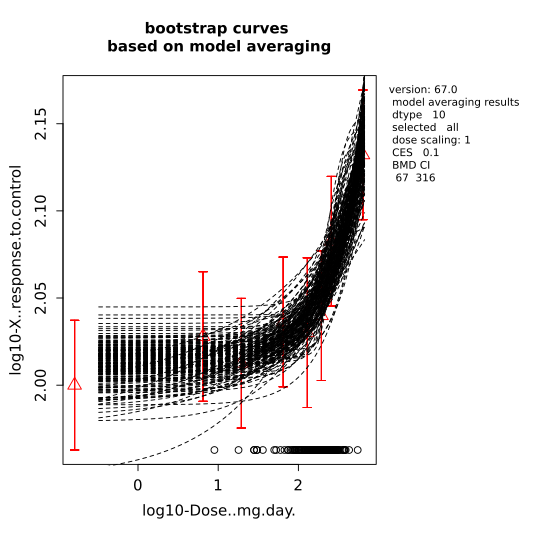


**Table S6** Results from a BMD analysis of the predicted dose-response curve (f_u,p_ values of 0.034) for human cardiotoxicity of methadone, using PBK modelling-based reverse dosimetry of hiPSC-CM MEA data. The table and figures present the characteristics of fitted models, the weights for model averaging and the final benchmark dose for 10% effect with the 95% lower–upper confidence limit values of the benchmark dose (BMDL_10_-BMDU_10_)

| model | converged | loglik | npar | AIC | weights | **Final BMD values from averaging** | |
| --- | --- | --- | --- | --- | --- | --- | --- |
|  |  |  |  |  |  | **BMDL_10_ (mg/day)** | **BMDU_10_ (mg/day)** |
| full model | yes | 37.87 | 9 | -57.74 | - | **109** | **509** |
| null model | yes | 22.47 | 2 | -40.94 | - |  |  |
| Expon. m3- | yes | 35.93 | 4 | -63.86 | 0.26 |  |  |
| Expon. m5- | yes | 36.26 | 5 | -62.52 | - |  |  |
| Hill m3- | yes | 35.93 | 4 | -63.86 | 0.26 |  |  |
| Hill m5- | yes | 36.17 | 5 | -62.34 | - |  |  |
| Inv.Expon. m3- | yes | 35.88 | 4 | -63.76 | 0.24 |  |  |
| Inv.Expon. m5- | yes | 36.18 | 5 | -62.36 | - |  |  |
| LN m3- | yes | 35.90 | 4 | -63.80 | 0.24 |  |  |
| LN m5- | yes | 36.20 | 5 | -62.40 | - |  |  |


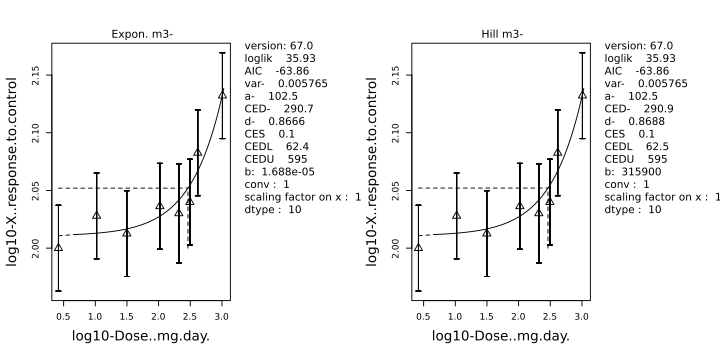

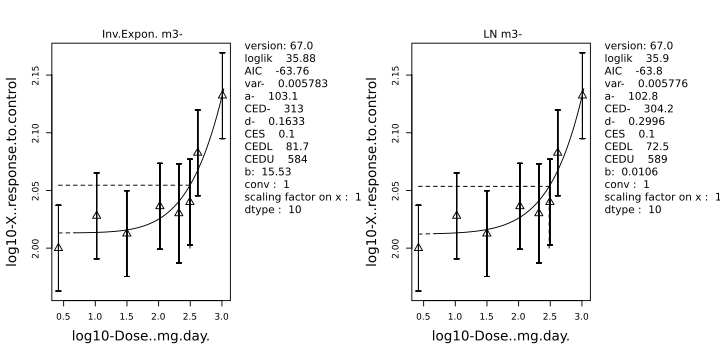

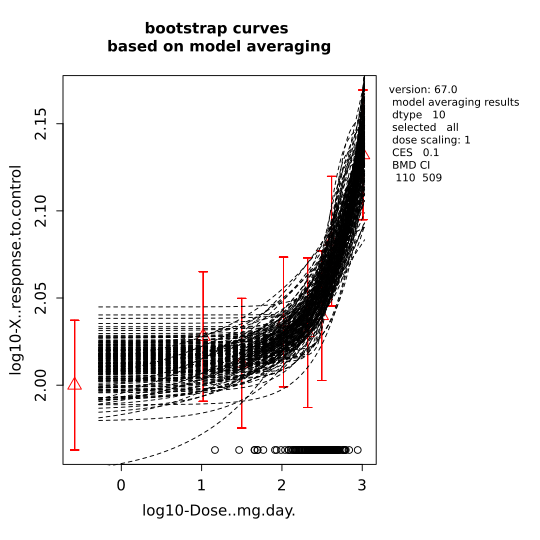


**Table S7** BMD_10_ values for cardiotoxicity of methadone predicted with the PBK modelling-based reverse dosimetry approach and therapeutic doses

|  | f_u,p_=0.034 | f_u,p_=0.055 | f_u,p_=0.15 | f_u,p_=0.22 |
| --- | --- | --- | --- | --- |
| BMDL_10_ (mg/day) | 109 | 66.7 | 24.2 | 16.5 |
| BMDU_10_ (mg/day) | 509 | 316 | 117 | 79.5 |
| initial dose for opioid-native patients (mg/day) ^a b^ | 10 | | | |
| initial dose for opioid-user (mg/day) ^a b^ | 30 | | | |
| maintenance dose (mg/day) ^b^ | 60-120 | | | |

^a^ Chou et al. 2014. ^b^ British Columbia Centre on Substance Use (BCCSU) 2017

**Supplementary materials 2**

;PBK model code human model

;=============================================================================

; physiological parameters

;=============================================================================

; tissue volumes (L or Kg)

BW = 70 ; body weight human in kg (Brown et al. 1997)

; all fractions taken from Brown et al. (1997)

VLc = 0.0257 ; fraction of liver tissue

VFc = 0.2142 ; fraction of fat tissue

VLuc = 0.0076 ; fraction of lung tissue

VAc = 0.0198 ; fraction of arterial blood: 0.074*1/4

VVc = 0.0593 ; fraction of venous blood: 0.074*3/4

VKc = 0.004 ; fraction of kidney tissue

VHc = 0.0047 ; fraction of heart tissue

VRc = 0.09-VLc - VLuc - VKc - VHc ; fraction of rapidly perfused tissue

VSc = 0.746-VFc ; fraction of blood flow to slowly perfused tissue

; total of fractions = 0.9151

VL = VLc * BW ; volume of liver

VF = VFc * BW ; volume of fat

VLu = VLuc * BW ; volume of lung

VK = VKc * BW ; volume of kidney

VH = VHc * BW ; volume of heart

VR = VRc * BW ; volume of rapidly perfused tissue

VS = VSc * BW ; volume of slowly perfused tissue

VA = VAc * BW ; volume of arterial blood

VV = VVc * BW ; volume of venous blood

;---------------------------------------------------------------------------------------------------------------------------------------

; blood flow rates (L/h)

QC = 15 * BW^0.74 ; QC = 15 * BW^0.74 (Brown et al. 1997)

QLc = 0.227 ; fraction of blood flow to liver

QFc = 0.052 ; fraction of blood flow to fat

QKc = 0.175 ; fraction of blood flow to kidney

QHc = 0.04 ; fraction of blood flow to heart

QSc = 0.24-QFc ; fraction of blood flow to slowly perfused tissue

QRc = 0.76-QLc-QKc-QHc ; fraction of blood flow to rapidly perfused tissue

; total of fractions = 1

; all fractions taken from Brown et al. (1997)

QL = QLc*QC ; blood flow rate to liver in L/hr

QF = QFc*QC ; blood flow rate to fat

QK = QKc * QC ; blood flow rate to kidney

QH = QHc*QC ; blood flow rate to heart

QR = QRc*QC ; blood flow rate to rapidly perfused tissue

QS = QSc*QC ; blood flow rate to slowly perfused tissue

;=============================================================================

; partition coefficients

;=============================================================================

; Tissue: blood partition coefficients of methadone and EDDP were obtained by dividing tissue: plasma partition coefficients by the corresponding blood/plasma ratio (BPr) reported in the study of Hsu et al. (2013) in which The BPr values of methadone and EDDP are 0.7 and 0.87, respectively. The tissue: plasma partition coefficients of methadone and EDDP were calculated using prediction method 1 which apply the algorithms of Berezhkovskiy (2004) in the Simcyp Simulator V18 Release 1 (Certara, Sheffield, UK).

PLmet = 12.448 ; liver/blood partition coefficient methadone

PFmet = 0.461 ; fat/blood partition coefficient methadone

PRmet = 12.448 ; rapidly perfused tissues/blood partition coefficient methadone

PSmet = 7.668 ; slowly perfused tissues/blood partition coefficient methadone

PLumet = 1.768 ; lung/blood partition coefficient methadone

PKmet = 7.555 ; kidney/blood partition coefficient methadone

PHmet = 4.904 ; heart/blood partition coefficient methadone

PLeddp = 11.51 ; liver/blood partition coefficient EDDP

PFeddp = 0.18 ; fat/blood partition coefficient EDDP

PReddp = 11.51 ; rapidly perfused tissues/blood partition coefficient EDDP

PSeddp = 7.06 ; slowly perfused tissues/blood partition coefficient EDDP

PLueddp = 1.56 ; lung/blood partition coefficient EDDP

PKeddp = 6.95 ; kidney/blood partition coefficient EDDP

PHeddp =4.48 ; heart/blood partition coefficient EDDP

;=============================================================================

; biochemical parameters

;=============================================================================

ka = 0.59 ; absorption rate constant (/h) obtained from Foster et al. (2000) and Wolff et al. (2000)

Fa = 0.88 ; fraction absorbed obtained from Ke et al. (2014)

RCLmet =1.45 ; renal clearance (l/h) of methadone is the average value obtained from Boulton et al. (2001); Foster et al. (2000) and Kharasch et al. (2009).

RCLeddp =19.99 ; renal clearance (l/h) of EDDP was obtained by the curve fit function of in vivo data (De Vos et al. 1995)

Kbile=1.64883 ; biliary excretion rate constant (/h) of EDDP was obtained by fit function of in vivo data (De Vos et al. 1995)

;--------------------------------------------------------------------------------------------------------------------------------------------------

; metabolism liver (methadone to EDDP)

; scaling factors

MPL=32 ; liver microsomal protein yield (mg/gram liver) (Barter et al. 2007)

L=VLc*1000 ; liver weight (gram/kg BW)

; metabolites of methadone, unscaled maximum rate of metabolism (nmol/mg protein/min) (in vitro incubation)

Vmaxc = 0.8156 ; obtained from in vitro microsomal incubation in the current study.

; metabolites of methadone, scaled maximum rate of metabolism (umol/h)

Vmax = Vmaxc / 1000 * 60 * MPL * L * BW

; metabolites of methadone, affinity constants (umol/L)

Km = 275 ; obtained from in vitro microsomal incubation in the current study.

;==========================================================================================

; run settings

;==========================================================================================

; molecular weight (g/mol)

MWmet= 309.4 ; molecular weight methadone

MWeddp = 277.41 ; molecular weight EDDP

; given dose (mg/kg bw) and oral dose in umol/kg bw

TDOSE = 40 ; whole body total dose in mg

GDOSE = TDOSE / BW ; GDOSE = given dose in mg per kg bw

ODOSE = GDOSE * 1e-3 / MWmet*1e6 ; determine odose (umol/kg bw)

DOSE = ODOSE * BW ; determine dose in umol

dose_int = 24 ; dosing interval in hours

; time (hrs)

Starttime = 0

Stoptime = 30*24 ; in hrs (days * hours in a day)

DTMIN = 1e-6

DTMAX = 1

DTOUT = 0

TOLERANCE = 0.00001

;==========================================================================================

; kinetics methadone

;==========================================================================================

; slowly perfused tissue compartment

; ASmet = Amount methadone in slowly perfused tissue, umol

ASmet' = QS * (CAmet - CVSmet)

Init ASmet = 0

CSmet = ASmet / VS

CVSmet = CSmet / PSmet

;---------------------------------------------------------------------------------------------------------------------------------------------------

; rapidly perfused tissue compartment

;ARmet = Amount methadone in rapidly perfused tissue, umol

ARmet' = QR * (CAmet - CVRmet)

Init ARmet = 0

CRmet = ARmet / VR

CVRmet = CRmet / PRmet

;---------------------------------------------------------------------------------------------------------------------------------------------------

; fat compartment

;AFmet = Amount methadone in fat tissue, umol

AFmet' = QF * (CAmet - CVFmet)

Init AFmet = 0

CFmet = AFmet / VF

CVFmet = CFmet / PFmet

;---------------------------------------------------------------------------------------------------------------------------------------------------

; uptake methadone from GI tract

;AGImet = Amount methadone remaining in GI tract ,umol

Init AGImet = 0

AGImet' = pulse(DOSE* Fa, 0, dose_int) + AGImet * -Ka

;---------------------------------------------------------------------------------------------------------------------------------------------------

;liver compartment

;ALmet = Amount methadone in liver tissue, umol

ALmet' = QL * (CAmet - CVLmet )+ (AGImet * Ka) - AMLmet'

Init ALmet = 0

CLmet = ALmet / VL

CVLmet = CLmet / PLmet

;AMLmet=Amount methadone metabolized in liver to EDDP

AMLmet' = (Vmax*CVLmet) / (Km + CVLmet)

init AMLmet = 0

;---------------------------------------------------------------------------------------------------------------------------------------------------

; kidney compartment

;AKmet = Amount methadone in kidney tissue, umol

AKmet' = QK * (CAmet - CVKmet)- ACLmet'

Init AKmet = 0

CKmet = AKmet / VK

CVKmet = CKmet / PKmet

;ACLmet=Amount methadone cleared renally

ACLmet'=RCLmet*CVKmet

init ACLmet = 0

;---------------------------------------------------------------------------------------------------------------------------------------------------

;heart compartment

;AHmet = Amount methadone in heart tissue, umol

AHmet' = QH * (CAmet - CVHmet)

Init AHmet = 0

CHmet = AHmet / VH

CVHmet = CHmet / PHmet

;---------------------------------------------------------------------------------------------------------------------------------------------------

;lung compartment

;ALumet = Amount methadone in lung tissue, umol

ALumet' = QC * (CVmet - CALumet)

Init ALumet = 0

CLumet = ALumet / VLu

CALumet = CLumet / PLumet

;---------------------------------------------------------------------------------------------------------------------------------------------------

; arterial blood compartment

;CAmet = Concentration arterial blood methadone

AAmet' = QC * (CALumet- CAmet);

Init AAmet = 0

CAmet= AAmet / VA

;------------------------------------------------------------------------------------------------------------------------------------------------------------------------------------

; venous blood compartment

;AVmet = amount venous blood methadone, umol

AVmet' = (QF * CVFmet + QR * CVRmet + QS * CVSmet + QL * CVLmet + QK * CVKmet + QH *CVHmet- QC * CVmet)

Init AVmet = 0

CVmet = (AVmet / VV)

;==========================================================================================

; Kinetics EDDP sub-model

;==========================================================================================

;slowly perfused tissue compartment

;ASeddp = Amount EDDP in slowly perfused tissue, umol

ASeddp' = QS * (CAeddp- CVSeddp)

Init ASeddp = 0

CSeddp = ASeddp / VS

CVSeddp = CSeddp / PSeddp

;---------------------------------------------------------------------------------------------------------------------------------------------------

; rapidly perfused tissue compartment

;AReddp = Amount EDDP in rapidly perfused tissue, umol

AReddp' = QR * (CAeddp - CVReddp)

Init AReddp = 0

CReddp = AReddp / VR

CVReddp= CReddp/ PReddp

;---------------------------------------------------------------------------------------------------------------------------------------------------

;fat compartment

;AFeddp= Amount EDDP in fat tissue, umol

AFeddp' = QF * (CAeddp - CVFeddp)

Init AFeddp= 0

CFeddp= AFeddp/ VF

CVFeddp = CFeddp/ PFeddp

;---------------------------------------------------------------------------------------------------------------------------------------------------

;liver compartment

;ALeddp= Amount EDDP in liver tissue, umol

ALeddp' = QL * (CAeddp - CVLeddp )+ AMLmet' -Abeddp'

Init ALeddp = 0

CLeddp = ALeddp / VL

CVLeddp = CLeddp / PLeddp

;Abeddp= biliary excretion

Abeddp'=Kbile*ALeddp

init Abeddp = 0

;---------------------------------------------------------------------------------------------------------------------------------------------------

;kidney compartment

;AKeddp = Amount EDDP in kidney tissue, umol

AKeddp' =QK * (CAeddp - CVKeddp) - ACLeddp'

Init AKeddp = 0

CKeddp = AKeddp / VK

CVKeddp= CKeddp / PKeddp

;ACLeddp=Amount EDDP cleared renally

ACLeddp'=RCLeddp*CVKeddp

init ACLeddp = 0

;---------------------------------------------------------------------------------------------------------------------------------------------------

;heart compartment

;AHeddp = Amount EDDP in heart tissue, umol

AHeddp' = QH * (CAeddp- CVHeddp)

Init AHeddp = 0

CHeddp = AHeddp / VH

CVHeddp = CHeddp / PHeddp

;--------------------------------------------------------------------------------------------------------------------------------------------------

;lung compartment

;ALueddp = Amount EDDP in lung tissue, umol

ALueddp' = QC * (CVeddp - CALueddp)

Init ALueddp = 0

CLueddp = ALueddp / VLu

CALueddp = CLueddp / PLueddp

;---------------------------------------------------------------------------------------------------------------------------------------------------

; arterial blood compartment

;CAeddp= Concentration arterial blood EDDP, umol

AAeddp' = QC * (CALueddp- CAeddp)

Init AAeddp = 0

CAeddp= AAeddp / VA

;---------------------------------------------------------------------------------------------------------------------------------------------------

; venous blood compartment

;AVeddp = amount venous blood EDDP, umol

AVeddp' = (QF * CVFeddp + QR * CVReddp+ QS * CVSeddp+ QL * CVLeddp + QK * CVKeddp + QH *CVHeddp- QC * CVeddp)

Init AVeddp = 0

CVeddp= (AVeddp/ VV)

;==========================================================================================

; mass balance calculations of methadone

;==========================================================================================

TotalMet' = pulse(DOSE *Fa, 0, dose_int)

init TotalMet = 1E-50

CalculatedMet = AFMet + ASMet+ ARMet + ALMet+ AVMet+ AAMet + AGIMet + AMLMet + ALuMet + AKMet + AHMet + ACLMet

ERRORmet = ((Totalmet - Calculatedmet) / (Totalmet + 1E-30)) * 100

MASSBALmet = Totalmet - Calculatedmet + 1

;==========================================================================================

; mass balance calculations of EDDP

;==========================================================================================

Totaleddp = AMLmet

Calculatededdp = AFeddp + ASeddp+ AReddp + ALeddp + AVeddp+ AAeddp+ ALueddp + AKeddp + AHeddp+ ACLeddp +Abeddp

ERROReddp= ((Totaleddp - Calculatededdp) / (Totaleddp + 1E-30)) * 100

MASSBALeddp = Totaleddp - Calculatededdp + 1

;==========================================================================================

; calculation with model

;==========================================================================================

; calculations for evaluation model performance methadone

CVmetW = (AVMet * (MWmet)) / VV ; concentration of methadone in venous blood (ug/L)

AUC' = CVmetW ; calculate AUC for methadone

init AUC = 0

CVheartmet= CVHmet*MWmet ; concentration of methadone in heart venous blood (ug/L)

;---------------------------------------------------------------------------------------------------------------------------------------------

; calculations for EDDP

CVeddpW = (AVeddp * (MWeddp)) / VV ; concentration of EDDP in venous blood (ug/L)

AUCeddp' = CVeddpW ; calculate AUC for EDDP blood

init AUCeddp = 0

CVhearteddp= CVHeddp*MWeddp ; concentration of EDDP in heart venous blood (ug/L)

**References**

Bart G, Wyman Z, Wang Q, Hodges JS, Karim R, Bart BA (2017) Methadone and the QTc interval: paucity of clinically significant factors in a retrospective cohort Journal of addiction medicine 11:489

Barter ZE et al. (2007) Scaling factors for the extrapolation of in vivo metabolic drug clearance from in vitro data: reaching a consensus on values of human micro-somal protein and hepatocellularity per gram of liver Current drug metabolism 8:33-45

Berezhkovskiy LM (2004) Determination of volume of distribution at steady state with complete consideration of the kinetics of protein and tissue binding in linear pharmacokinetics Journal of pharmaceutical sciences 93:364-374

Boulton DW, Arnaud P, DeVane CL (2001) Pharmacokinetics and pharmacodynamics of methadone enantiomers after a single oral dose of racemate Clinical Pharmacology & Therapeutics 70:48-57

British Columbia Centre on Substance Use (BCCSU) (2017) A Guideline for the Clinical Management of Opioid Use Disorder. https://www.bccsu.ca/wp-content/uploads/2017/06/BC-OUD-Guidelines_June2017.pdf. Accessed 20 November 2019

Brown RP, Delp MD, Lindstedt SL, Rhomberg LR, Beliles RP (1997) Physiological parameter values for physiologically based pharmacokinetic models Toxicology and industrial health 13:407-484

Carlquist JF et al. (2015) A possible mechanistic link between the CYP2C19 genotype, the methadone metabolite ethylidene-1, 5-dimethyl-3, 3-diphenylpyrrolidene (EDDP), and methadone-induced corrected QT interval prolongation in a pilot study Molecular diagnosis & therapy 19:131-138

Chang KC et al. (2012) Gender‐specific differences in susceptibility to low‐dose methadone‐associated QTc prolongation in patients with heroin dependence Journal of cardiovascular electrophysiology 23:527-533

Chou R et al. (2014) Methadone safety: a clinical practice guideline from the American Pain Society and College on Problems of Drug Dependence, in collaboration with the Heart Rhythm Society The Journal of Pain 15:321-337

Chowdhury M, Wong J, Cheng A, Khilkin M, Palma E (2015) Methadone Therapy in Underserved Urban Community: QT c Prolongation and Life‐Threatening Ventricular Arrhythmias Cardiovascular therapeutics 33:127-133

Cruciani RA et al. (2005) Measurement of QTc in patients receiving chronic methadone therapy Journal of pain and symptom management 29:385-391

De Vos J, Ufkes J, van Wilgenburg H, Geerlings P, van den Brink W (1995) Pharmacokinetics of methadone and its primary metabolite in 20 opiate addicts European journal of clinical pharmacology 48:361-366

Eap CB et al. (2007) Stereoselective block of hERG channel by (S)‐methadone and QT interval prolongation in CYP2B6 slow metabolizers Clinical Pharmacology & Therapeutics 81:719-728

Ehret GB et al. (2006) Drug-induced long QT syndrome in injection drug users receiving methadone: high frequency in hospitalized patients and risk factors Archives of internal medicine 166:1280-1287

Esses JL, Rosman J, Do LT, Schweitzer P, Hanon S (2008) Successful transition to buprenorphine in a patient with methadone-induced torsades de pointes Journal of interventional cardiac electrophysiology 23:117-119

Fareed A, Vayalapalli S, Scheinberg K, Gale R, Casarella J, Drexler K (2013) QTc interval prolongation for patients in methadone maintenance treatment: a five years follow-up study The American journal of drug and alcohol abuse 39:235-240

Foster DJ, Somogyi AA, Dyer KR, White JM, Bochner F (2000) Steady‐state pharmacokinetics of (R)‐and (S)‐methadone in methadone maintenance patients British journal of clinical pharmacology 50:427-440

Fredheim OMS, Borchgrevink PC, Hegrenæs L, Kaasa S, Dale O, Klepstad P (2006) Opioid switching from morphine to methadone causes a minor but not clinically significant increase in QTc time: A prospective 9-month follow-up study Journal of pain and symptom management 32:180-185

Heesch CB, Copfer AE, Davis SJ, Edwards BW (2015) Evaluation of Methadone-Induced QTc Prolongation in a Veteran Population Federal Practitioner 32:36

Hsu Y-C et al. (2013) Methadone concentrations in blood, plasma, and oral fluid determined by isotope-dilution gas chromatography–mass spectrometry Analytical and bioanalytical chemistry 405:3921-3928

Kharasch ED, Walker A, Whittington D, Hoffer C, Bedynek PS (2009) Methadone metabolism and clearance are induced by nelfinavir despite inhibition of cytochrome P4503A (CYP3A) activity Drug and alcohol dependence 101:158-168

Krantz MJ, Lewkowiez L, Hays H, Woodroffe MA, Robertson AD, Mehler PS (2002) Torsade de pointes associated with very-high-dose methadone Annals of internal medicine 137:501-504

Krantz MJ, Lowery CM, Martell BA, Gourevitch MN, Arnsten JH (2005) Effects of methadone on QT‐interval dispersion Pharmacotherapy: The Journal of Human Pharmacology and Drug Therapy 25:1523-1529

Maremmani I, Pacini M, Cesaroni C, Lovrecic M, Perugi G, Tagliamonte A (2005) QTc interval prolongation in patients on long-term methadone maintenance therapy European addiction research 11:44-49

Martell BA, Arnsten JH, Krantz MJ, Gourevitch MN (2005) Impact of methadone treatment on cardiac repolarization and conduction in opioid users The American journal of cardiology 95:915-918

Peles E, Bodner G, Kreek MJ, Rados V, Adelson M (2007) Corrected‐QT intervals as related to methadone dose and serum level in methadone maintenance treatment (MMT) patients—a cross‐sectional study Addiction 102:289-300

Reddy S, Hui D, Osta BE, de la Cruz M, Walker P, Palmer JL, Bruera E (2010) The effect of oral methadone on the QTc interval in advanced cancer patients: a prospective pilot study Journal of palliative medicine 13:33-38

Roy AK, McCarthy C, Kiernan G, McGorrian C, Keenan E, Mahon NG, Sweeney B (2012) Increased incidence of QT interval prolongation in a population receiving lower doses of methadone maintenance therapy Addiction 107:1132-1139

Wedam EF, Bigelow GE, Johnson RE, Nuzzo PA, Haigney MC (2007) QT-interval effects of methadone, levomethadyl, and buprenorphine in a randomized trial Archives of internal medicine 167:2469-2475

Wolff K, Rostami‐Hodjegan A, Hay A, Raistrick D, Tucker G (2000) Population‐based pharmacokinetic approach for methadone monitoring of opiate addicts: potential clinical utility Addiction 95:1771-1783
